# Complexity and Dynamics of the Winemaking Bacterial Communities in Berries, Musts, and Wines from Apulian Grape Cultivars through Time and Space

**DOI:** 10.1371/journal.pone.0157383

**Published:** 2016-06-14

**Authors:** Marinella Marzano, Bruno Fosso, Caterina Manzari, Francesco Grieco, Marianna Intranuovo, Giuseppe Cozzi, Giuseppina Mulè, Gaetano Scioscia, Gabriel Valiente, Apollonia Tullo, Elisabetta Sbisà, Graziano Pesole, Monica Santamaria

**Affiliations:** 1 Institute of Biomembranes and Bioenergetics, CNR, Bari, Italy; 2 Institute of Sciences of Food Production, CNR, Operative Unit of Lecce, Lecce (Le), Italy; 3 Department of Biosciences, Biotechnology and Biopharmaceutics, University of Bari “A. Moro” Bari, Italy; 4 Institute of Sciences of Food Production, CNR, Bari, Italy; 5 GBS BAO Advanced Analytics Services and MBLab, IBM, Bari, Italy; 6 Algorithms, Bioinformatics, Complexity and Formal Methods Research Group, Polytechnic University of Catalonia, Barcelona, Spain; 7 Institute for Biomedical Technologies, CNR, Bari, Italy; Wilfrid Laurier University, CANADA

## Abstract

Currently, there is very little information available regarding the microbiome associated with the wine production chain. Here, we used an amplicon sequencing approach based on high-throughput sequencing (HTS) to obtain a comprehensive assessment of the bacterial community associated with the production of three Apulian red wines, from grape to final product. The relationships among grape variety, the microbial community, and fermentation was investigated. Moreover, the winery microbiota was evaluated compared to the autochthonous species in vineyards that persist until the end of the winemaking process. The analysis highlighted the remarkable dynamics within the microbial communities during fermentation. A common microbial core shared among the examined wine varieties was observed, and the unique taxonomic signature of each wine appellation was revealed. New species belonging to the genus *Halomonas* were also reported. This study demonstrates the potential of this metagenomic approach, supported by optimized protocols, for identifying the biodiversity of the wine supply chain. The developed experimental pipeline offers new prospects for other research fields in which a comprehensive view of microbial community complexity and dynamics is desirable.

## Introduction

In recent years, several investigations have been conducted to characterize the microbiome associated with different ecosystems, such as natural niches, agricultural and industrial environments, and plant and animal hosts. An understanding of this wide biodiversity is fundamental not only for ecological purposes, as a key to maintaining a healthy environment and sustainable economy, but also for human health, revealing the crucial role of microbes in diseases onset [1, 2] and food safety [3, 4]. In particular, the growing consumer demand for safeguarding of food products and components has strongly encouraged the development of new tools and approaches for investigating the taxonomical and functional complexity of microbial communities in order to assess their contribution to food quality, safety, and traceability. Although microbes play important roles in human nutrition, much remains to be explored because the vast majority of these microbes cannot be cultured by standard techniques [5, 2] (i.e., plate isolation, enrichment, and cultivation of single strains). Furthermore, such classical methods are typically labour-intensive and costly [6], and are limited for processing large numbers of samples, for which comparison is often the only key to identifying unique traits or common trends.

Winemaking is a composite process in which numerous microorganisms, mainly yeast and bacteria, play important roles. Yeast promote alcoholic fermentation (AF), converting the fermentable sugars to ethanol and carbon dioxide, whereas lactic acid bacteria (LAB) carry out malolactic fermentation (MLF). MLF is the conversion of L-malic acid to L-lactic acid and carbon dioxide [7]. MLF biological transformation is highly recommended for the production of some white wines and nearly all red wines, because it enhances the microbiological stability of the product [8, 9] and improves its organoleptic properties [10, 11]. Knowing the composition and population dynamics of the microbial consortia throughout vinification is fundamental for controlling the process and improving the quality and safety of the final product [12, 13, 14]. In fact, despite the number of studies on the microorganisms associated with fermentation, especially fungi, in California, South Africa, and New Zealand [15, 16, 17], there is a poor understanding of the bacterial community as a whole, its dynamics throughout the fermentation chain, and its correlation with wine appellation or geographic origin. In fact, the “winemaking habitat” represents an emblematic case study since it could be described as a dynamic network of microbial populations and biochemical flow.

Recently developed culture-independent methods, such as metagenomic approaches based on the extraction of all the genetic material from a selected habitat and subsequent sequencing and bioinformatic analyses, provide an unprecedented opportunity to enlarge the detectable biodiversity of microbial communities. Large-scale sequencing of the entire metagenome (shotgun approach) and selective screening of particular species markers (target-oriented or amplicon sequencing approach), mainly one or more hypervariable regions of the 16S rRNA gene for bacteria identification, are made possible through the use of high-throughput sequencing (HTS) platforms, which are the only feasible instruments for handling the rapid production of millions of sequences from multiple biological samples.

Using amplicon sequencing approaches to characterize the microbiome associated with wine fermentation is a particularly problematic, as a large number of compounds in the wine “habitat” can alter the quality and efficiency of microbial nucleic acid extraction and thus negatively affect subsequent processing (e.g., Taq polymerase activity) [18, 19]. To date, only a few HTS-based studies on the dynamics of the microbial population in wine production have been published [20], and no Illumina 16S amplicon-based sequencing of red wine fermentation, which includes spontaneous MLF, has been reported.

The goal of this study was to describe the taxonomic complexity and evolution of the bacterial community throughout the production of typical Apulian wines, in particular, Cabernet, Negramaro, and Primitivo, by investigating the bacterial composition from grape to the end of MLF, a fermentative process that consistently shapes the organoleptic properties of the wine and can adversely affect quality due to the production of undesirable metabolites [21, 22]. It is widely accepted that autochthonous microbes are an important source of the distinctive metabolites that influence the chemical profile and flavour of wine [23, 20, 24].

Cabernet Sauvignon is an international grape cultivar; although it is associated with the Bordeaux region of France, it is grown all over the world. Cabernet Sauvignon variety is “the world’s most renowned grape variety for the production of fine red wine” [25]. Negramaro is a non-aromatic red wine grape cultivar originally from southeast Italy that produces wines with a pleasurable organoleptic bouquet largely appreciated by consumers. Negramaro wine has a dark red colour, and it combines a peculiar aroma with earthy bitterness. Negramaro has economic value because it is the essential grape cultivar in the production of 14 protected designation of origin (PDO) Italian wines [26, 27]. Primitivo cv. (*Vitis vinifera* L.) is an early, strong wine grape cultivar that is widely grown throughout the Apulia region in southern Italy. Primitivo is an economically important cultivar because it is the main grape variety for the production of several PDO wines. Primitivo grapes produce a varietal wine characterized by high alcoholic and tannic notes, with a ruby-purple colour and a spicy, red-fruit aroma [28].

Here, we describe, for the first time, a comprehensive assessment of the winemaking microbiome using an amplicon sequencing metagenomic approach optimized to drastically reduce the technical difficulties of dealing with a very challenging matrix like must and aimed at characterizing and comparing the diversity throughout production and among different Apulian wine appellations.

## Materials and Methods

### Sampling

Grape musts (*gm*), belonging to three wine grape varieties, Cabernet (C), Negramaro (N), and Primitivo (P), were collected throughout fermentation using grapes from the Tormaresca winery fields located in San Pietro Vernotico (Apulia, Italy).

Must samples were collected in duplicate, corresponding to grapes from two adjacent rows of vines, named 1 and 2, and at five different times during fermentation, as described below:

Time 0 (T0) (sample name *sAF*), after grape crushing and before adding exogenous starter yeasts
T1 (*24hAF*), 24 hours after starter addition (*Saccharomyces cerevisiae*)
T2 (*sMLF*), end of AF (sugar level, 2 g/L), which corresponds to the start of spontaneous MLF
T3 (*hMLF*), half way through spontaneous MLF; and
T4 (*eMLF*), end of spontaneous MLF (malic acid = 0).

In order to characterize the resident winery microbiota, healthy grape bunches belonging to the three wine grape varieties under investigation were collected, and the microbiota was isolated from grapes by washing 24 bunches in sterile distilled water (1:1 weight/volume) on a rotary shaker at 100 rpm for 5 min. The samples were centrifuged at 12,000 rpm for 12 min, and the pellet was recovered and suspended in 10 mL of sterile distilled water. The grape must (*gm*) samples and the bunch washing water (*bww*) were stored at -20°C until DNA extraction.

Tormaresca winery gave us permission to collect the grapes and corresponding must samples. The experimental procedures used in the study did not require specific permission and did not involve endangered or protected species.

### DNA extraction

Ten millilitres of *gm* or *bww* were centrifuged (30 min, 10,000 × *g*, 4°C), and the pellet was washed in 2 mL of TE buffer (10 mM Tris HCl, 1 mM EDTA, pH 8.0). After a second centrifugation (10,000 × *g* for 15 min at 4°C), the supernatant was discarded, and the pellet was dissolved in 300 μL of TE. Subsequently, total genomic DNA was isolated using the FastDNA SPIN kit for soil (BIO 101, Carlsbad, CA) according to the manufacturer’s instructions. Cell lysis was achieved by bead beating in a FastPrep Instrument (BIO 101) at speed 6 for 40 s. The quantity and quality of extracted DNA were assessed by spectrophotometry (Eppendorf, Hamburg, Germany) and agarose gel (1%) electrophoresis, respectively.

### 16S rRNA library preparation and sequencing

An amplicon-based approach was applied to the DNA extracted from the tested samples for prokaryotic identification. Of the nine hyper-variable regions present in the 16S rRNA gene, the V5–V6 regions were chosen as amplification targets. Amplicon libraries were prepared using 5 ng of metagenomic DNA extracted from each sample. The strategy used to prepare the 16S rRNA amplicon-based library is described in detail in Manzari et al. [29]. Equimolar quantities of the purified amplicons were pooled and subjected to 2 × 250 bp paired-end sequencing on the Illumina MiSeq platform. To increase the genetic diversity, as required by the MiSeq platform, a phage PhiX genomic DNA library was added to the mix and co-sequenced. The run was performed in duplicate (named A and B).

### Taxonomic analysis

To characterize the prokaryotic composition of the tested samples, a stand-alone version of the Bioinformatic analysis of Metagenomic AmpliconS (BioMaS) pipeline [30] was used to analyse the obtained MiSeq paired-end (PE) reads. Overlapping PE reads were merged into consensus sequences with Flash [31]. These were treated to remove sequences shorter than 50 bp, and they were then dereplicated with Usearch [32] while retaining information about the total number of original consensus sequences. Non-overlapping PE reads were cleaned by removing low-quality regions (quality-score threshold = 25) and discarding PE reads containing sequences shorter than 50 nt using Trim-Galore.

Subsequently, to minimize background noise due to host DNA contamination, which, due to its abundance, could adversely affect the bacterial identification, leading to biased results, both consensus and non-overlapping denoised PE reads were mapped against a collection of *Vitis vinifera* mitochondrial and plastidial 16S reference sequences using Bowtie2 [33]. Sequences with ≥97% identity were discarded.

To determine the taxonomic affiliations of the retained consensus and unmerged PE reads, they were compared to the Ribosomal Database Project II (RDP-II; release 11.2) [34] using Bowtie2. Mapping data were filtered according to two parameters: identity percentage and query coverage (≥95%). In particular, sequences that matched sequences in RDP-II, with at least 97% identity were directed to species classification [35], while those with 90–97% identity were classified at higher taxonomic levels.

Statistically significant differences in the bacterial taxonomic composition of the tested samples were determined at the family, genus, and species levels using DESeq2 [36] a R/Bioconductor package.

The alpha diversity index (Shannon Index, H-index) and the species richness estimator (Chao1) were calculated by using the R package phyloseq [37] from the operational taxonomic unit (OTU) matrix generated by QIIME as input [38]. The OTU matrix was calculated by summing the OTU counts derived from the two biological replicates for each sample. The H-indices obtained for each sample were analysed by a two-tailed t-test. Pairwise comparisons were made between time points and grape varieties. Finally, the R package Vegan [39] was used to generate rarefaction curves based on the OTU matrix.

Principal coordinate analysis (PCoA) was performed using the R package Vegan based on the Bray-Curtis dissimilarities calculated for the composition of the bacterial communities at the genus level.

## Results and Discussion

To characterize the prokaryotic microbiota associated with the winemaking process, grape musts (*gm*), belonging to three wine grape varieties, Cabernet (C), Negramaro (N), and Primitivo (P) grown in the Apulia Region of southern Italy, were sampled. The *gm* samples were collected at five time points during winemaking (*sAF*, *24hAF*, *sMLF*, *hMLF*, and *eMLF*, see Experimental Procedures for details) and subjected to DNA extraction. The extracted DNA samples were used as templates for library preparation and an amplicon sequencing approach was applied to characterize the composition of the prokaryotic community.

**Fig 1:**
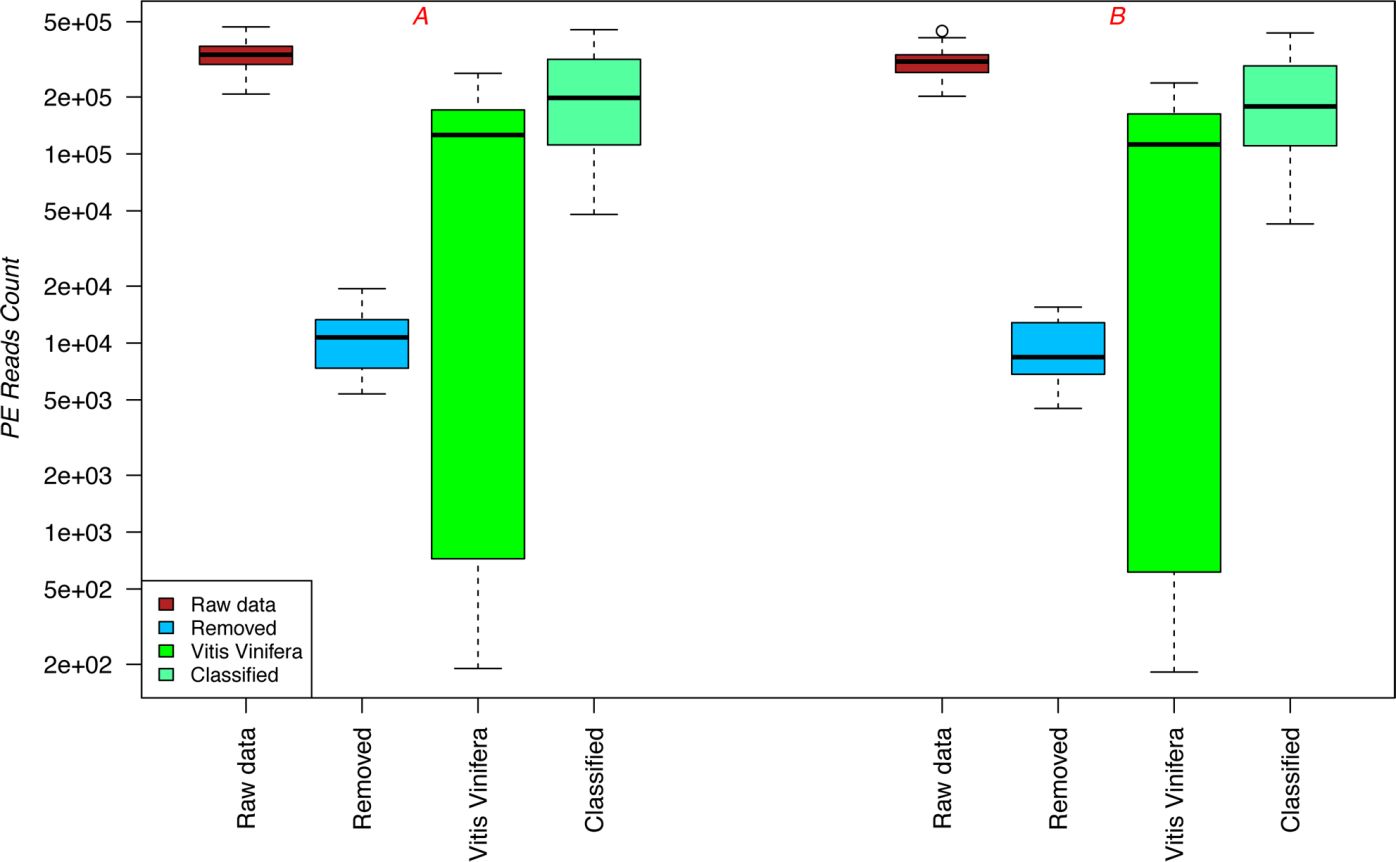
Graphical summary of the results obtained by applying the BioMaS pipeline. Box plot showing the number of PE reads that were produced by the MiSeq platform for both sequencing runs (raw data), including low quality reads removed after denoising, host reads mapped to *Vitis vinifera*, and bacterial reads taxonomically classified by BioMaS.

Libraries containing 420-bp dual indexed amplicons were successfully sequenced on the MiSeq platform using V2 2 × 250 bp PE sequencing chemistry. Approximately 10 million and 9 million PE reads were generated in the first (A) and second (B) runs, respectively (SRA accession number SRP072913). The median number of PE reads produced per sample was 334,000 and 308,000 for runs A and B, respectively (Fig 1). After adapter trimming and removing pairs containing reads shorter than 50 bp, the PE reads were analysed with BioMaS [30] (S1 Table). For both sequencing runs (A and B), the number of PE reads produced, including low-quality reads removed after denoising, host reads mapped to the *Vitis vinifera* genome, and bacterial reads taxonomically classified by BioMaS, are presented as box plots in Fig 1. The data obtained from the two sequencing runs was highly reproducible. For all three wine varieties tested, the number of PE reads that mapped to the host genome was related to the fermentation step; while the number of host-mapping PE reads was remarkable in the first steps of fermentation (*sAF*, 71.2%; *24hAF*, 43.8%; *sMLF*, 51.33%) it dramatically decreased in the later steps (*hMLF*, 0.33%; *eMLF*, 0.15%) (see S1 Table). This reduction at later time points could be due to progressive deterioration of *V*. *vinifera* cells and subsequent DNA degradation in the harsh fermentation environment (which is characterized by a low pH, the presence of degradative enzymes in the must, and elevated temperature) [40, 41, 42].

**Fig 2:**
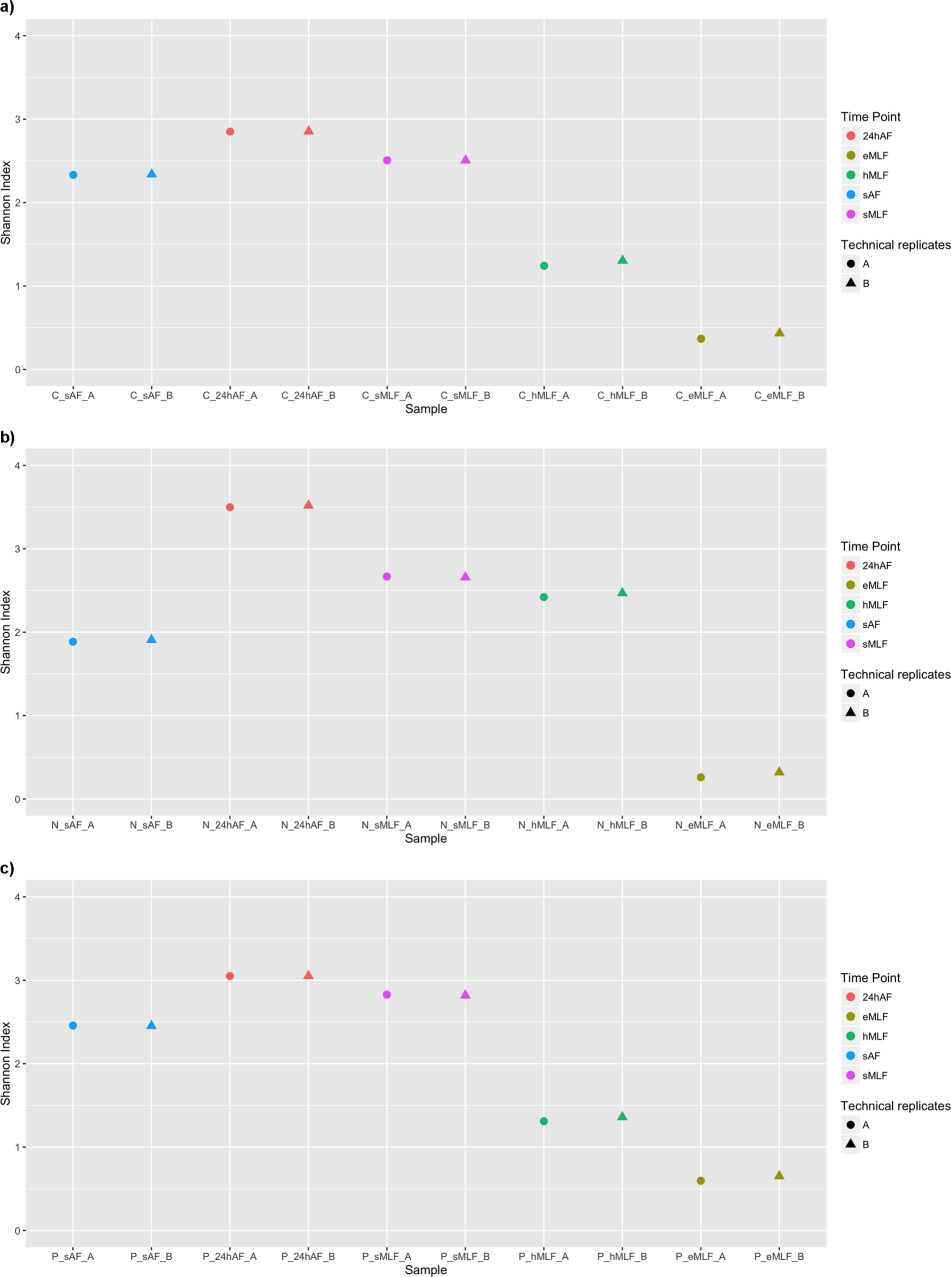
Scatter plot of the calculated Shannon index values. Shannon index values calculated based on the OTU matrix, obtained from the Illumina MiSeq raw data, are shown for all the tested wine varieties. Only the OTUs that were taxonomically labelled as bacterial or not mapped to *Vitis Vinifera* plastids were considered. The values calculated for each sample are plotted for Cabernet (a), Negramaro (b), and Primitivo (c). Different colours and shapes were used to distinguish the five time points (*sAF*, *24hAF*, *sMLF*, *hMLF*, and *eMLF*) and the technical replicates (sequencing runs A and B), respectively.

To obtain a preliminary snapshot of the microbial biodiversity in term of richness and abundance that might distinguish the analysed samples, the Shannon diversity index (H) was calculated for the raw data. The PE reads were clustered into operational taxonomic units (OTUs) by using QIIME [38], and for each sequencing run, the H index was calculated, as the sum of the two biological replicates (Fig 2). The biodiversity trends were similar for the three wine varieties, and the data from the two sequencing runs were highly reproducible. As shown in Fig 2, at the beginning of the winemaking process, 24 hours after starter addition (*24hAF*), there was a slight increase in the H indices compared to those recorded at *sAF*, followed by a reduction until the end of the process (*eMLF*). These results suggest that the bacterial population changed during vinification. The increased bacterial diversity at *24hAF* could be due to microbial contamination from the winery, as has been described by du Toit et al. [42] and Bokulich et al. [43]. In particular, both authors stated that bacterial strains could be isolated from the cellar environment, including barrels and winery equipment such as pipes and valves. These strains would be in addition to those residing on the grapes, which were detected at *sAF*. Moreover, the enhancement in bacterial diversity observed after starter addition could also be caused by the interaction between yeasts and bacteria, as was reported by du Toit et al. [42] and Lasik [44]. In particular, the *S*. *cerevisiae* starter strains could positively affect LAB growth and MLF as a result of mannoprotein production and essential nutrient release due to autolysis. In contrast, the decrease in the diversity of the bacterial population observed during fermentation (after starter addition) can be explained by the presence of different wild yeasts on the berry skin that are not sensitive to the SO_2_ that is routinely added as a fungicide at the beginning of the AF. Indeed, previous studies have indicated that wild yeast can exert antibacterial effects, and thus can modify the composition and dynamics of the bacterial community during fermentation [45, 46].

The statistical significance of differences between the H-indices of samples was evaluated by a two-tailed t-test. For each tested grape variety, the pairwise comparisons showed statistically significant differences (*p* < 0.05) between most time points, except *sAF* and *sMLF* for Cabernet, *sAF* and *hMLF* and *sMLF* and *hMLF* for Negramaro, and *hMLF* and *eMLF* for Primitivo (S2 Table). Moreover, the significance of differences was also evaluated for the three different grape varieties at each time point. The results are shown in S2 Table.

**Fig 3:**
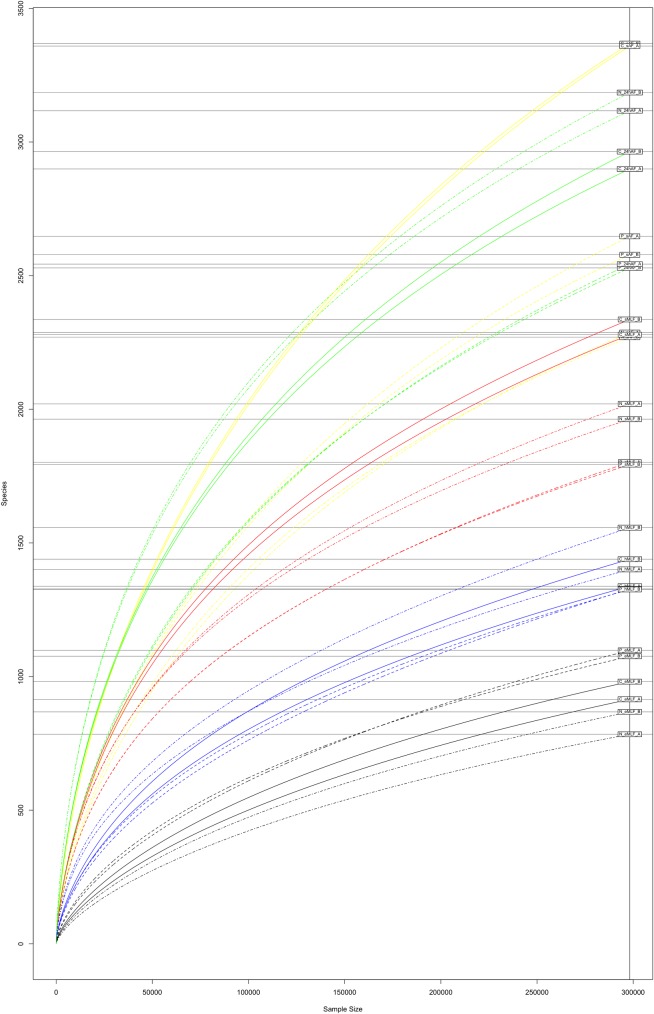
Rarefaction curves for the tested samples. Rarefaction curves calculated using the OTU matrix obtained from the Illumina MiSeq raw data by using QIIME are shown for all the analysed samples and sequencing runs. The five time points are shown in different colours: *sAF* (yellow), *24hAF* (green), *sMLF* (red), *hMLF* (blue), and *eMLF* (black), and the wine varieties Cabernet, Negramaro, and Primitivo are shown as solid, dot-dash, and dashed lines, respectively.

To estimate the expected species richness of the tested samples, the Chao 1 indices were calculated based on the OTU matrix generated by QIIME (Table 1). As expected, the estimated richness decreased after starter addition and remained low until the end of fermentation, showing a trend similar to that of the Shannon indices. The Chao1 index values ranged from 1342.64 to 5006.81. By comparing the number of observed OTUs and the Chao 1 values, we were able to calculate the coverage (%) in terms of species richness achieved in our assay (Table 1). Good coverage of the prokaryotic community was achieved, with values ranging from 50% to 74%. These data were confirmed by the rarefaction curves obtained for each sample and sequencing run (Fig 3).

**Table 1:** The number of observed OTUs, the species richness estimator (Chao 1), and coverage (calculated from the ratio of observed OTUs and Chao 1) obtained for each sample and sequencing run (A or B).

Sample	Observed OTUs	Chao 1	Coverage (%)
C_sAF_A	3359	5006.81	67.09%
C_sAF_B	3368	4858.52	69.32%
C_24hAF_A	2899	3974.13	72.95%
C_24hAF_B	2964	4142.89	71.54%
C_sMLF_A	2279	3356.61	67.90%
C_sMLF_B	2336	3567.19	65.49%
C_hMLF_A	1338	2185.25	61.23%
C_hMLF_B	1439	2427.53	59.28%
C_eMLF_A	915	1553.13	58.91%
C_eMLF_B	981	1614.42	60.76%
N_sAF_A	2287	3303.67	69.23%
N_sAF_B	2270	3117.01	72.83%
N_24hAF_A	3117	4186.32	74.46%
N_24hAF_B	3185	4463.29	71.36%
N_sMLF_A	2020	3052.02	66.19%
N_sMLF_B	1963	2898.58	67.72%
N_hMLF_A	1401	2293.61	61.08%
N_hMLF_B	1557	2726.65	57.10%
N_eMLF_A	784	1342.64	58.39%
N_eMLF_B	868	1541.60	56.31%
P_sAF_A	2647	3749.22	70.60%
P_sAF_B	2578	3476.18	74.16%
P_24hAF_A	2543	3816.09	66.64%
P_24hAF_B	2529	3790.78	66.71%
P_sMLF_A	1802	2879	62.59%
P_sMLF_B	1793	2675.55	67.01%
P_hMLF_A	1328	2650.03	50.11%
P_hMLF_B	1326	2247.18	59.01%
P_eMLF_A	1098	1866.08	58.84%
P_eMLF_B	1076	1814.32	59.31%

The taxonomic assignment of sequences was performed using BioMaS. The PE sequences matching those in the RDP-II database (96.45% of sequences in run A and 96.55% of sequences in run B) exceeding the 90% identity threshold were classified and assigned to a clade in the NCBI taxonomy (S3 Table). The remaining reads (approximately 3%) that did not show any significant match in the reference database were not assigned to any taxonomic clade. Those reads are likely derived from novel taxa not yet identified and represented in RDP-II. For both sequencing runs, about 96% and 98% of the classified sequences were assigned at the family and genus levels, respectively, whereas about 76% of the PE reads were assigned at the species level.

**Fig 4:**
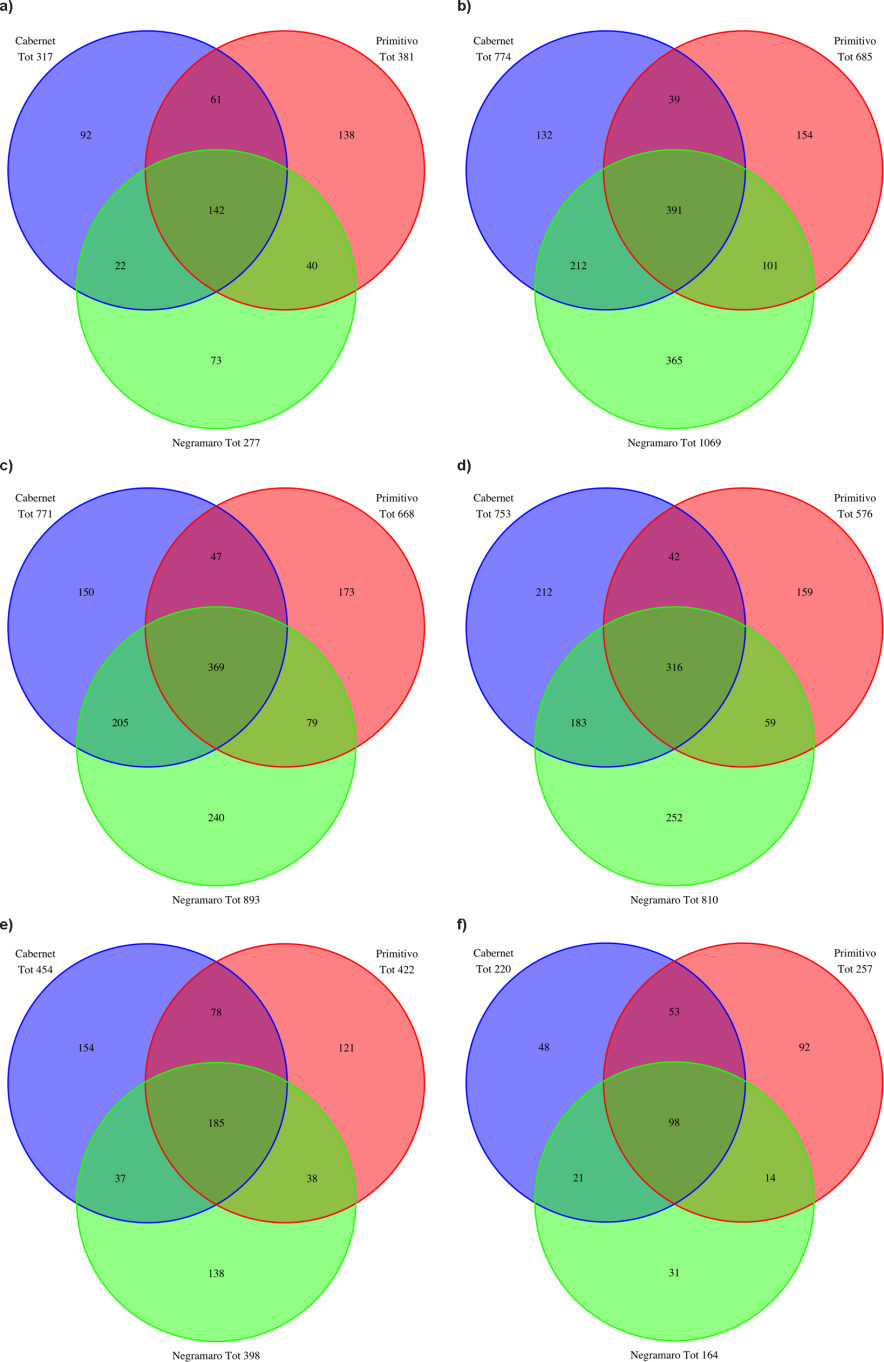
Venn diagrams showing the number of common and unique species detected in the tested wine varieties. Venn diagrams showing the number of species detected in the three analysed wines (Cabernet, Negramaro, and Primitivo) at each time point: *sAF* (a), *24hAF* (b), *sMLF* (c), *hMLF* (d), and *eMLF* (e). (f) Venn diagram showing the number of species detected in samples from the three wines throughout fermentation (from *sAF* to *eMLF*).

For each time point, the number of species identified for the three wine varieties was evaluated and graphically represented as a Venn diagram (Fig 4). For all three varieties, the number of identified taxa changed during fermentation. At the first time point (*sAF*), 317, 277, and 381 species were identified in C, N, and P, respectively. At the *24hAF* time point, the number of taxa increased considerably compared to that at *sAF* (Fig 4A and 4B). From the beginning of MLF (*sMLF*) until the end of fermentation (*eMLF*), a reduction in the number of taxa was observed for all three varieties (Fig 4C–4E). Another Venn diagram was drawn including only those species that were detected throughout the fermentation process (from *sAF* to *eMLF*; Fig 4F). A common taxonomic core of 98 species was observed among the three varieties. Each variety was characterized by a unique microbial biodiversity pattern. In particular, 48, 31, and 92 species were exclusively detected in the C, N, and P samples, respectively. The number of species common to two varieties are also shown in the diagram (Fig 4F).

**Fig 5:**
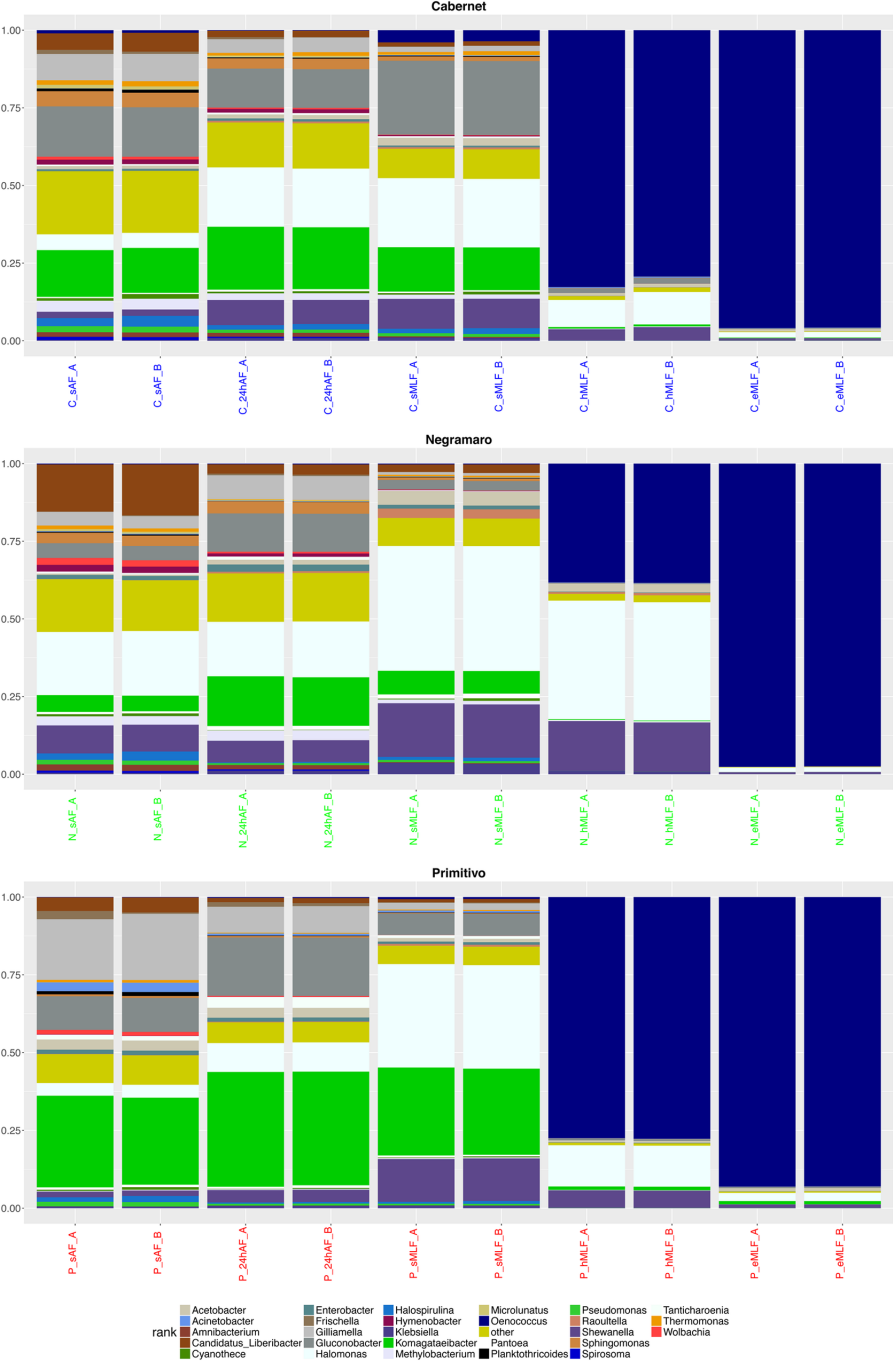
Stacked box-plot of relative taxa abundances. Stacked box-plot displaying the relative abundances of the taxa (≥1%). In particular, a specific plot was produced for each wine variety (Cabernet, Negramaro, and Primitivo) to describe the changes in genera abundance at the five time points (*sAF*, *24hAF*, *sMLF*, *hMLF*, and *eMLF*). Taxa for which the relative abundance was less than 1% were grouped and named “others” in the graph.

The taxa at the genus level with a relative abundance ≥1% [47] determined by summing the counts derived from the two biological replicates for each sample are plotted in Fig 5. As previously described, for all three varieties, there was an evident change in the taxa and their relative abundance during fermentation. At the beginning of the winemaking process (*sAF*), the genera that were common to all three varieties (with relative abundance ≥1%) were *Candidatus_Liberibacter* (average relative abundance 5.69% in C, 15.81% in N, and 4.59% in P), *Gilliamella* (8.61% in C, 4.02% in N, and 20.39% in P), *Gluconobacter* (16.06% in C, 4.69% in N, and 10.86% in P), *Halomonas* (4.95% in C, 20.59% in N, and 4.12% in P), *Halospirulina* (3.05% in C, 2.48% in N, and 1.58% in P), *Komagataeibacter* (14.76% in C, 5.24% in N, and 28.70% in P), *Pseudomonas* (1.90% in C, 1.43% in N, and 1.44% in P), and *Shewanella* (2.03% in C, 8.83% in N, and 1.78% in P). Statistically significant differences (adjusted *p* < 0.05) were evaluated by comparing the normalized abundance values obtained for each variety. In particular, *Candidatus_Liberibacter*, *Halomonas*, and *Shewanella* were more abundant in N than in C and P, while *Gilliamella*, *Gluconobacter*, and *Komagataeibacter* were more abundant in P than in N and C. For *Halospirulina* and *Pseudomonas*, no significant differences were detected among the varieties. In addition to the above mentioned genera (shown in Fig 5), *Amnibacterium* (average relative abundance: 1.48% in C and 1.99% in N), *Methylobacterium* (3.50% in C and 2.95% in N), *Hymenobacter* (1.53% in C and 2.26% in N), *Sphingomonas* (4.77% in C and 3.33% in N), and *Thermomonas* (1.58% in C and 1.12% in N) were observed both in C and N, *Enterobacter* (1.34% in N and 1.40% in P) and *Wolbachia* (2.13% in N and 1.32% in P) were detected in N and P, and *Acetobacter* (1.03% in C and 3.25% in P) and *Frischella* (1.03% in C and 1.51% in P) were detected in C and P. Moreover, *Cyanothece* (average relative abundance, 1.17%), *Microlunatus* (1.60%), and *Spirosoma* (1%) were observed in C, while *Acinetobacter* (2.89%), *Planktothricoides* (1.23%), and *Tanticharoenia* (1.52%) were detected in P. These genera were also detected in the other wine varieties; however, their relative abundance was less than 1%. In Fig 5, the taxa for which the relative abundance was less than 1% were grouped and called “other,” which corresponded to 648, 556, and 594 genera in C, N, and P, respectively. Concerning the different bacterial genera identified during AF, the analyses showed a predominance of species belonging to *Gluconobacter*, a genus of acetic acid bacteria that are typically associated with berry skin [11], in the microbiome of the three wines. This finding is in agreement with the results of similar studies using culture-independent methods conducted in Spain [48], the USA [20], and Slovakia [49]. In addition to these dominant genera, a number of other species belonging to several other genera, which were considered innocuous environmental contaminants [50], were detected during AF. Several of these genera, including *Pseudomonas*, *Enterobacter*, *Acinetobacter*, *Pantoea*, *Methylobacterium*, *Gilliamella*, and *Amnibacterium*, have been identified in other similar studies [43, 49, 20], whereas a number of novel genera, such as *Candidatus_Liberibacter*, *Frischella*, *Halomonas*, *Hymenobacter*, *Komagataeibacter*, *Microlunatus*, *Planktothricoides*, *Shewanella*, *Spirosoma*, *Tanticharoenia*, *Thermomonas*, and *Wolbachia* were, to the best of our knowledge, uniquely associated with the wine habitat.

For the three wine varieties, the number of identified taxa gradually decreased in subsequent winemaking steps. In the last two steps of MLF (*hMLF* and *eMLF*), the same taxa were identified in the C, N, and P samples, although with different relative abundances. In particular, *Shewanella* (average relative abundance: 3.95% in C, 15.95% in N, and 5.54% in P), *Halomonas* (9.59% in C, 38.13% in N, and 13.22% in P), and *Oenococcus* (81.02% in C, 38.33% in N, and 77.60% in P) were abundant at the *hMLF* time point, while at the *eMLF* time point, *Oenococcus* was the predominant genus (95.83% in C, 97.53% in N, and 93.02% in P) followed by *Halomonas* (1.81% in C, 1.51% in N, and 2.64% in P). At the species level, at the end of fermentation (*eMLF*) only two taxa were detected in the samples: *Oenococcus oeni*, which was the most abundant (96.08% in C, 97.69% in N, and 93.78% in P), and *Halomonas phoecae* (1.81% in C, 1.49% in N, and 2.66% in P). Thus, these two species showed different population dynamics during the winemaking process; while the abundance of *O*. *oeni* increased during fermentation, reaching the highest values at the end of the process (*eMLF*), *H*. *phoecae* decreased after the start of spontaneous MLF. Regarding the bacterial genera identified during MLF, the obtained data were partially consistent with those reported by Renouf et al. [51] and Ruiz et al. [48], who both described *Oenococcus* as the predominant genus. In contrast, species belonging to the genus *Halomonas* have never been reported in wines, although *Halomonas* species have been isolated from diverse high-osmolarity habitats [52] and recently from traditional Korean fermented food [53].

**Fig 6:**
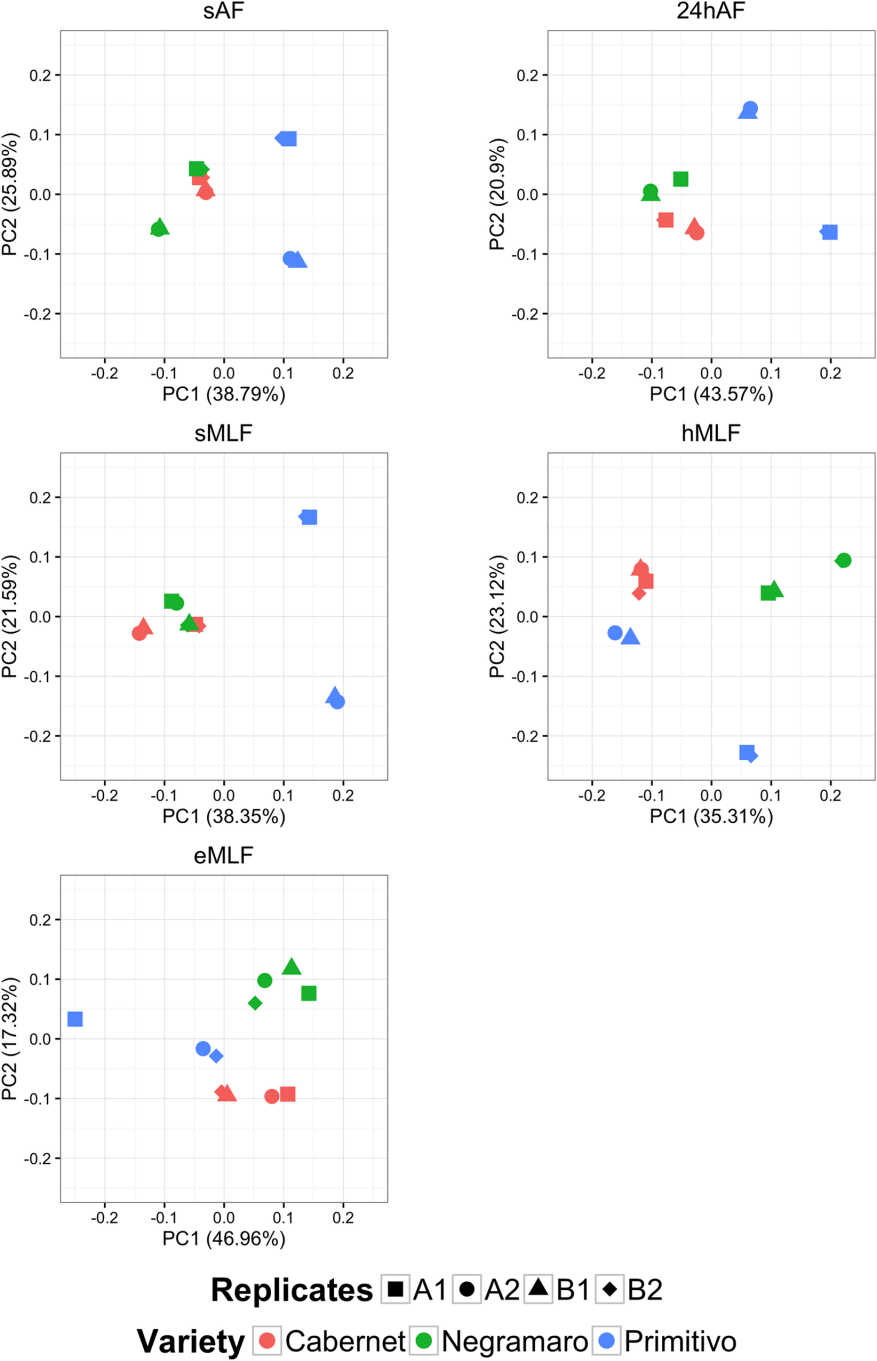
Principal coordinate analysis plots generated at the genus level for the five fermentation time points. Principal coordinate analysis (PCoA) plots based on the taxonomical classification at the genus level are shown for the five tested time points: *sAF*, *24hAF*, *sMLF*, *hMLF*, and *eMLF*. Biological replicates (1 and 2) and sequencing runs (A and B) of the three analysed wines (Cabernet, Negramaro and Primitivo) are all shown in each plot as different shapes.

To investigate the relationships among the microbial community, fermentation step, and grape variety, a multivariate statistical procedure, called principal coordinate analysis (PCoA), was carried out. At the genus level, the PCoA plot showed a clear separation of the bacterial populations between C, N, and P during the winemaking process (Fig 6). This result was particularly evident at the end of MLF (*eMLF*), where three separate groups were observed. Furthermore, the PCoA component values calculated for each sample supported clear reproducibility of the composition of the microbial communities between biological replicates (1 and 2) and sequencing runs (A and B). Different profiles were only observed between the biological replicates of P samples, which were taxonomically distant. In particular, at *eMLF* replicate 2 was taxonomically closer to the other two wine varieties, C and N, than to its biological replicate (replicate 1). These data are probably related to the conditions unique to that particular row of wine grapes. In fact, considering the data obtained from Tormaresca winery, at *eMLF*, the pH and alcohol values (3.35 and 12.10°, respectively) recorded for replicate 1 of P were lower than those obtained for replicate 2 (3.47 and 14.77°, respectively) and for the other varieties (3.45 and 13.3° for C_1, 3.55 and 13.62° for C_2, 3.48 and 13.55° for N_1, and 3.43 and 14.31° for N_2, respectively).

The last aspect investigated was microbial “contamination” from the winery that, as previously described [43], seems to have a key role in the specific organoleptic features of the final product. To examine this, healthy grape bunches belonging to the three tested wine grape varieties were collected from the same supply chains as the *gm* samples, the DNA was extracted from bunch washing water samples (*bww*) (see Materials and Methods), and the amplicon libraries were sequenced together with the *gm* samples on the MiSeq. The PE reads from the *bww* samples were processed by the BioMaS pipeline, and the results were compared to those obtained from the *gm* samples at the five time points. For this analysis, the taxa that were absent from the field (*bww*) samples but present during fermentation (from *sAF* to *eMLF*) were included.

Surprisingly, winery-specific microflora were detected in each variety. In particular, at the genus level, *Micrococcus* was identified in the C samples, *Haemophilus*, *Luteimonas*, and *Wautersiella* were observed in the N samples, and *Actinomyces*, *Alistipes*, *Ameyamaea*, *Aurantimonas*, *Caenimonas*, *Cellvibrio*, *Dietzia*, *Empedobacter*, *Leifsonia*, *Micrococcus*, *Nevskia*, *Pediococcus*, *Ruminococcus*, *Tolumonas*, *Tsukamurella*, and *Wautersiella* were detected in the P samples. At the species level, 3, 10, and 28 winery species were exclusively observed in the C, N, and P samples, respectively (S4 Table). Conversely, the number of species that originated in the field and persisted throughout the fermentation process was 229 in C, 157 in N, and 244 in P (S5 Table).

In the present study, we described a large-scale exploration of the winemaking microbiome using an amplicon sequencing approach optimized to drastically reduce the technical difficulties associated with a very challenging matrix like must, aimed at characterizing and comparing microbial diversity along the production chain and across different Apulian wine appellations. A comprehensive assessment of the composition of the bacterial community was investigated by sequencing the V5–V6 hypervariable region of 16S rRNA using the MiSeq (Illumina) platform to determine if i) a common microbial core is shared between different wine varieties, ii) a taxonomic signature could be assigned to each appellation, iii) contamination from winery microflora remarkably affects microbial composition, iv) which of the vineyard autochthonous species persist until the end of the winemaking chain, and v) if there are microorganisms that have never been described in association with winemaking. In particular, the results were compared both horizontally (at the different steps of winemaking) and vertically (between varieties) using both test-hypothesis and multivariate statistical analyses to investigate the mutual relationships among the microbial community, fermentation step, and grape variety.

## Conclusions

This study demonstrated the amazing potential of a metagenomic approach based on HTS technologies to identify the large number of species involved in the production of wine, which was clearly underestimated. Indeed, most of the species identified have never been reported, and based on the obtained data, it can be assumed that the detectable biodiversity will further increase. In fact, a considerable number of microorganisms, which can be observed but not identified, may be soon classified thanks to the continuous enrichment of reference molecular databases. Our survey might also have crucial implications in the context of purely practical aspects, such as controls and improvement of the quality and safety of the final product [54], its appreciation by the consumer, and its commercial value. Moreover, the developed experimental pipeline provides intriguing prospects for many other research fields in which a comprehensive view of microbial complexity and dynamics is desirable.

## Supporting Information

S1 Table Analysis of sequencing data using BioMaS. Data related to each bioinformatics analysis step are provided for the analysed samples. In particular, (i) Sample Name = label assigned to the analysed sample, (ii) Grape Variety; (iii) Time Point: step in the wine fermentation process; (iv) Biological Replicate: Samples were collected twice, and the two biological replicates were labelled 1 and 2; (v) Technical Replicate: Each sample was sequenced twice, and the sequencing runs were labelled A and B; (vi) PE = number of produced paired-end (PE) reads; (vii) Merged = number of merged pairs generated by Flash; (viii) % Merged = percentage of merged pairs, relative to the number of produced PE reads; (ix) Unmerged = number of unmerged pairs; (x) % Unmerged = percentage of unmerged pairs, relative to the number of produced PE reads; (vi) Removed = number of removed pairs; (xii) % Removed = percentage of removed pairs, relative to the number of produced PE reads; (xiv) *Vitis vinifera* = number of sequences mapped to a collection of *Vitis vinifera* mitochondrial and plastidial 16S references and removed; (xv) % *Vitis*: percentage of removed sequences, relative to the number of merged and unmerged PE reads; (xvi) Classified = number of classified pairs, (xvii) % Classified = percentage of classified pairs, relative to the sequences that passed the denoising and host mapping steps (Merged + Unmerged–*Vitis vinifera*).
(DOCX)

S2 Table Pairwise comparisons between time points and grape varieties. Two-tailed t-tests were used to compare the H-indices obtained for each sample. In particular, pairwise comparisons were made between each pair of time points for a given variety (a) and between pairs of varieties at a given time point (b). Statistically significant differences are indicated with asterisks (*p < 0.1, **p < 0.05, and ***p < 0.01).
(DOCX)

S3 Table Taxonomic assignments determined by BioMaS. Data related to the obtained taxonomic classification. In particular, (i) Sample Name = label assigned to the analysed sample, (ii) Classified = number of classified paired-end (PE) reads, (iii) Kingdom: PE reads classified at the kingdom level, (iv) % Kingdom: percentage of PE reads assigned at the kingdom level, relative to the total number of assigned PE reads, (v) Class: PE reads classified at the class level, (vi) % Class: percentage of PE reads assigned at the class level, relative to the total number of assigned PE reads, (vii) Order: PE reads classified at the order level, (viii) % Order: percentage of PE reads assigned at the order level, relative to the total number of assigned PE reads, (ix) Family: PE reads classified at the family level, (x) % Family: percentage of PE reads assigned at the family level, relative to the total number of assigned PE reads, (xi) Genus: PE reads classified at the genus level, (xii) % Genus: percentage of PE reads assigned at the genus level, relative to the total number of assigned PE reads, (xiii) Species: PE reads classified at the species level, (xiv) % Species: percentage of PE reads assigned at the species level, relative to the total number of assigned PE reads.
(DOCX)

S4 Table Winery microflora associated with the tested wine varieties. List of taxa at the genus and species levels identified in the Cabernet, Negramaro, and Primitivo samples that were not detected in the field (the *bww* samples) but were present during fermentation from start (*sAF*) to finish (*eMLF*).
(DOCX)

S5 Table Microflora associated with the tested wine varieties that originated in the field and persisted throughout fermentation. List of the species that originated in the field and persisted throughout the fermentation process, from start (*sAF*) to finish (*eMLF*).
(DOCX)
