# Supplementary material for: Complexity and Dynamics of the Winemaking Bacterial Communities in Berries, Musts, and Wines from Apulian Grape Cultivars through Time and Space
Source: PLoS One. 2016 Jun 14;11(6):e0157383. doi: 10.1371/journal.pone.0157383 (PMC4907434; doi:10.1371/journal.pone.0157383)
Supplement: S1 Table — Data related to each bioinformatics analysis step are provided for the analysed samples. In particular, (i) Sample Name = label assigned to the analysed sample, (ii) Grape Variety; (iii) Time Point: step in the wine fermentation process; (iv) Biological Replicate: Samples were collected twice, and the two biological replicates were labelled 1 and 2; (v) Technical Replicate: Each sample was sequenced twice, and the sequencing runs were labelled A and B; (vi) PE = number of produced paired-end (PE) reads; (vii) Merged = number of merged pairs generated by Flash; (viii) % Merged = percentage of merged pairs, relative to the number of produced PE reads; (ix) Unmerged = number of unmerged pairs; (x) % Unmerged = percentage of unmerged pairs, relative to the number of produced PE reads; (vi) Removed = number of removed pairs; (xii) % Removed = percentage of removed pairs, relative to the number of produced PE reads; (xiv) Vitis vinifera = number of sequences mapped to a collection of Vitis vinifera mitochondrial and plastidial 16S references and removed; (xv) % Vitis: percentage of removed sequences, relative to the number of merged and unmerged PE reads; (xvi) Classified = number of classified pairs, (xvii) % Classified = percentage of classified pairs, relative to the sequences that passed the denoising and host mapping steps (Merged + Unmerged–Vitis vinifera). (DOCX) [file pone.0157383.s001.docx]

**S1 Table**. **Sequencing data analysis performed by BioMaS.**

| **Sample Name** | **Grape Variety** | **Time Point** | **Biological Replicate** | **Technical Replicate** | **PE** | **Merged** | **% Merged** | **Unmerged** | **% Unmerged** | **Removed** | **% Removed** | **Vitis Vinifera** | **% Vitis** | **Classifed** | **%Classifed** |
| --- | --- | --- | --- | --- | --- | --- | --- | --- | --- | --- | --- | --- | --- | --- | --- |
| C_sAF_A_1 | Cabernet | sAF | 1 | A | 357,919 | 335,071 | 93.62% | 6,345 | 1.77% | 16,503 | 4.61% | 251,355 | 73.62% | 80,712 | 89.62% |
| C_sAF_A_2 | Cabernet | sAF | 2 | A | 407,086 | 383,172 | 94.13% | 7,328 | 1.80% | 16,586 | 4.07% | 266,810 | 68.33% | 111,645 | 90.26% |
| C_24hAF_A_1 | Cabernet | 24hAF | 1 | A | 225,545 | 212,769 | 94.34% | 3,356 | 1.49% | 9,420 | 4.18% | 112,607 | 52.10% | 99,424 | 96.05% |
| C_24hAF_A_2 | Cabernet | 24hAF | 2 | A | 269,466 | 254,215 | 94.34% | 4,283 | 1.59% | 10,968 | 4.07% | 149,914 | 57.99% | 102,509 | 94.41% |
| C_sMLF_A_1 | Cabernet | sMLF | 1 | A | 310,138 | 288,283 | 92.95% | 5,999 | 1.93% | 15,856 | 5.11% | 139,120 | 47.27% | 150,771 | 97.17% |
| C_sMLF_A_2 | Cabernet | sMLF | 2 | A | 401,870 | 382,070 | 95.07% | 6,779 | 1.69% | 13,021 | 3.24% | 255,425 | 65.69% | 127,641 | 95.67% |
| C_hMLF_A_1 | Cabernet | hMLF | 1 | A | 382,439 | 373,057 | 97.55% | 2,771 | 0.72% | 6,611 | 1.73% | 524 | 0.14% | 372,625 | 99.29% |
| C_hMLF_A_2 | Cabernet | hMLF | 2 | A | 207,225 | 199,081 | 96.07% | 2,414 | 1.16% | 5,730 | 2.77% | 1,901 | 0.94% | 197,879 | 99.14% |
| C_eMLF_A_1 | Cabernet | eMLF | 1 | A | 276,011 | 268,866 | 97.41% | 1,767 | 0.64% | 5,378 | 1.95% | 195 | 0.07% | 268,519 | 99.29% |
| C_eMLF_A_2 | Cabernet | eMLF | 2 | A | 368,424 | 359,107 | 97.47% | 2,550 | 0.69% | 6,767 | 1.84% | 380 | 0.11% | 358,774 | 99.31% |
| N_sAF_A_1 | Negramaro | sAF | 1 | A | 292,734 | 276,645 | 94.50% | 4,657 | 1.59% | 11,432 | 3.91% | 224,810 | 79.92% | 47,855 | 84.71% |
| N_sAF_A_2 | Negramaro | sAF | 2 | A | 339,220 | 317,278 | 93.53% | 5,537 | 1.63% | 16,405 | 4.84% | 261,473 | 81.00% | 55,181 | 89.96% |
| N_24hAF_A_1 | Negramaro | 24hAF | 1 | A | 360,956 | 338,243 | 93.71% | 6,294 | 1.74% | 16,419 | 4.55% | 141,134 | 40.96% | 197,727 | 97.21% |
| N_24hAF_A_2 | Negramaro | 24hAF | 2 | A | 407,570 | 380,954 | 93.47% | 7,247 | 1.78% | 19,369 | 4.75% | 170,896 | 44.02% | 208,596 | 95.99% |
| N_sMLF_A_1 | Negramaro | sMLF | 1 | A | 267,803 | 255,196 | 95.29% | 4,193 | 1.57% | 8,414 | 3.14% | 156,147 | 60.20% | 100,138 | 96.99% |
| N_sMLF_A_2 | Negramaro | sMLF | 2 | A | 330,288 | 312,886 | 94.73% | 5,681 | 1.72% | 11,721 | 3.55% | 168,564 | 52.91% | 145,597 | 97.06% |
| N_hMLF_A_1 | Negramaro | hMLF | 1 | A | 358,304 | 346,165 | 96.61% | 3,523 | 0.98% | 8,616 | 2.40% | 742 | 0.21% | 346,144 | 99.20% |
| N_hMLF_A_2 | Negramaro | hMLF | 2 | A | 343,509 | 331,064 | 96.38% | 5,084 | 1.48% | 7,361 | 2.14% | 723 | 0.22% | 332,736 | 99.20% |
| N_eMLF_A_1 | Negramaro | eMLF | 1 | A | 357,381 | 347,681 | 97.29% | 2,369 | 0.66% | 7,331 | 2.05% | 190 | 0.05% | 347,704 | 99.38% |
| N_eMLF_A_2 | Negramaro | eMLF | 2 | A | 324,695 | 316,293 | 97.41% | 2,356 | 0.73% | 6,046 | 1.86% | 336 | 0.11% | 316,365 | 99.39% |
| P_sAF_A_1 | Primitivo | sAF | 1 | A | 315,129 | 299,862 | 95.16% | 4,585 | 1.45% | 10,682 | 3.39% | 163,662 | 53.76% | 133,246 | 94.65% |
| P_sAF_A_2 | Primitivo | sAF | 2 | A | 283,853 | 269,256 | 94.86% | 4,148 | 1.46% | 10,449 | 3.68% | 198,650 | 72.66% | 68,141 | 91.15% |
| P_24sAF_A_1 | Primitivo | 24hAF | 1 | A | 297,381 | 273,782 | 92.06% | 5,744 | 1.93% | 17,855 | 6.00% | 86,930 | 31.10% | 187,035 | 97.11% |
| P_24hAF_A_2 | Primitivo | 24hAF | 2 | A | 440,708 | 421,371 | 95.61% | 6,056 | 1.37% | 13,281 | 3.01% | 163,842 | 38.33% | 259,012 | 98.27% |
| P_SMLF_A_1 | Primitivo | sMLF | 1 | A | 302,791 | 286,936 | 94.76% | 4,905 | 1.62% | 10,950 | 3.62% | 88,557 | 30.34% | 199,913 | 98.34% |
| P_sMLF_A_2 | Primitivo | sMLF | 2 | A | 370,779 | 355,993 | 96.01% | 5,041 | 1.36% | 9,745 | 2.63% | 190,920 | 52.88% | 166,951 | 98.14% |
| P_hMLF_A_1 | Primitivo | hMLF | 1 | A | 428,590 | 414,000 | 96.60% | 3,870 | 0.90% | 10,720 | 2.50% | 1,358 | 0.32% | 412,777 | 99.10% |
| P_hMLF_A_2 | Primitivo | hMLF | 2 | A | 319,172 | 305,921 | 95.85% | 4,011 | 1.26% | 9,240 | 2.89% | 568 | 0.18% | 306,588 | 99.10% |
| P_eMLF_A_1 | Primitivo | eMLF | 1 | A | 302,279 | 293,099 | 96.96% | 2,434 | 0.81% | 6,746 | 2.23% | 1,265 | 0.43% | 291,729 | 99.14% |
| P_eMLF_A_2 | Primitivo | eMLF | 2 | A | 469,354 | 453,896 | 96.71% | 3,805 | 0.81% | 11,653 | 2.48% | 296 | 0.06% | 453,582 | 99.16% |
| C_sAF_B_1 | Cabernet | sAF | 1 | B | 319,956 | 301,788 | 95.95% | 5,222 | 1.63% | 12,946 | 4.05% | 222,324 | 72.42% | 76,668 | 90.53% |
| C_sAF_B_2 | Cabernet | sAF | 2 | B | 358,317 | 338,160 | 96.22% | 6,610 | 1.84% | 13,547 | 3.78% | 233,439 | 67.71% | 100,810 | 90.55% |
| C_24hAF_B_1 | Cabernet | 24hAF | 1 | B | 201,605 | 189,351 | 95.83% | 3,846 | 1.91% | 8,408 | 4.17% | 99,349 | 51.42% | 90,079 | 95.98% |
| C_24hAF_B_2 | Cabernet | 24hAF | 2 | B | 288,041 | 269,163 | 95.50% | 5,922 | 2.06% | 12,956 | 4.50% | 157,865 | 57.39% | 110,387 | 94.17% |
| C_sMLF_B_1 | Cabernet | sMLF | 1 | B | 286,838 | 266,206 | 94.96% | 6,180 | 2.15% | 14,452 | 5.04% | 127,870 | 46.94% | 140,464 | 97.20% |
| C_sMLF_B_2 | Cabernet | sMLF | 2 | B | 356,512 | 339,515 | 96.97% | 6,205 | 1.74% | 10,792 | 3.03% | 224,819 | 65.03% | 116,132 | 96.06% |
| C_hMLF_B_1 | Cabernet | hMLF | 1 | B | 351,983 | 342,991 | 98.34% | 3,136 | 0.89% | 5,856 | 1.66% | 529 | 0.15% | 343,114 | 99.28% |
| C_hMLF_B_2 | Cabernet | hMLF | 2 | B | 331,922 | 320,530 | 97.76% | 3,963 | 1.19% | 7,429 | 2.24% | 2,951 | 0.91% | 318,806 | 99.15% |
| C_eMLF_B_1 | Cabernet | eMLF | 1 | B | 235,461 | 229,101 | 98.09% | 1,854 | 0.79% | 4,506 | 1.91% | 219 | 0.09% | 229,131 | 99.30% |
| C_eMLF_B_2 | Cabernet | eMLF | 2 | B | 334,265 | 325,485 | 98.22% | 2,814 | 0.84% | 5,966 | 1.78% | 409 | 0.12% | 325,529 | 99.28% |
| N_sAF_B_1 | Negramaro | sAF | 1 | B | 249,559 | 238,048 | 96.81% | 3,562 | 1.43% | 7,949 | 3.19% | 191,856 | 79.41% | 42,651 | 85.72% |
| N_sAF_B_2 | Negramaro | sAF | 2 | B | 311,241 | 290,810 | 95.27% | 5,696 | 1.83% | 14,735 | 4.73% | 237,037 | 79.94% | 53,584 | 90.10% |
| N_24hAF_B_1 | Negramaro | 24hAF | 1 | B | 320,051 | 301,991 | 96.06% | 5,440 | 1.70% | 12,620 | 3.94% | 124,859 | 40.61% | 177,651 | 97.30% |
| N_24hAF_B_2 | Negramaro | 24hAF | 2 | B | 378,570 | 356,116 | 95.91% | 6,977 | 1.84% | 15,477 | 4.09% | 157,095 | 43.27% | 198,193 | 96.21% |
| N_sMLF_B_1 | Negramaro | sMLF | 1 | B | 223,837 | 213,284 | 96.98% | 3,797 | 1.70% | 6,756 | 3.02% | 129,414 | 59.62% | 85,189 | 97.17% |
| N_sMLF_B_2 | Negramaro | sMLF | 2 | B | 323,967 | 304,730 | 96.05% | 6,430 | 1.98% | 12,807 | 3.95% | 162,661 | 52.28% | 143,858 | 96.87% |
| N_hMLF_B_1 | Negramaro | hMLF | 1 | B | 324,328 | 312,670 | 97.57% | 3,775 | 1.16% | 7,883 | 2.43% | 816 | 0.26% | 312,915 | 99.14% |
| N_hMLF_B_2 | Negramaro | hMLF | 2 | B | 303,765 | 290,113 | 97.31% | 5,487 | 1.81% | 8,165 | 2.69% | 615 | 0.21% | 292,030 | 99.00% |
| N_eMLF_B_1 | Negramaro | eMLF | 1 | B | 304,398 | 296,027 | 97.96% | 2,151 | 0.71% | 6,220 | 2.04% | 182 | 0.06% | 296,095 | 99.36% |
| N_eMLF_B_2 | Negramaro | eMLF | 2 | B | 297,490 | 288,897 | 98.04% | 2,769 | 0.93% | 5,824 | 1.96% | 415 | 0.14% | 289,306 | 99.33% |
| P_sAF_B_1 | Primitivo | sAF | 1 | B | 263,891 | 251,048 | 96.71% | 4,151 | 1.57% | 8,692 | 3.29% | 136,849 | 53.62% | 112,045 | 94.67% |
| P_sAF_B_2 | Primitivo | sAF | 2 | B | 257,806 | 244,877 | 96.57% | 4,095 | 1.59% | 8,834 | 3.43% | 178,850 | 71.84% | 63,994 | 91.26% |
| P_24sAF_B_1 | Primitivo | 24hAF | 1 | B | 253,071 | 237,867 | 95.56% | 3,960 | 1.56% | 11,244 | 4.44% | 74,783 | 30.92% | 162,654 | 97.37% |
| P_24hAF_B_2 | Primitivo | 24hAF | 2 | B | 411,449 | 390,740 | 96.45% | 6,088 | 1.48% | 14,621 | 3.55% | 149,951 | 37.79% | 241,985 | 98.02% |
| P_SMLF_B_1 | Primitivo | sMLF | 1 | B | 269,406 | 256,693 | 96.87% | 4,291 | 1.59% | 8,422 | 3.13% | 79,230 | 30.36% | 179,018 | 98.49% |
| P_sMLF_B_2 | Primitivo | sMLF | 2 | B | 331,341 | 317,731 | 97.39% | 4,965 | 1.50% | 8,645 | 2.61% | 169,142 | 52.42% | 150,672 | 98.12% |
| P_hMLF_B_1 | Primitivo | hMLF | 1 | B | 389,039 | 377,704 | 98.04% | 3,722 | 0.96% | 7,613 | 1.96% | 1,141 | 0.30% | 377,161 | 99.18% |
| P_hMLF_B_2 | Primitivo | hMLF | 2 | B | 283,058 | 273,177 | 97.58% | 3,042 | 1.07% | 6,839 | 2.42% | 406 | 0.15% | 273,603 | 99.20% |
| P_eMLF_B_1 | Primitivo | eMLF | 1 | B | 278,694 | 271,507 | 98.29% | 2,435 | 0.87% | 4,752 | 1.71% | 1,143 | 0.42% | 270,560 | 99.18% |
| P_eMLF_B_2 | Primitivo | eMLF | 2 | B | 447,332 | 436,175 | 98.28% | 3,478 | 0.78% | 7,679 | 1.72% | 252 | 0.06% | 436,279 | 99.29% |
