# Supplementary material for: Complexity and Dynamics of the Winemaking Bacterial Communities in Berries, Musts, and Wines from Apulian Grape Cultivars through Time and Space
Source: PLoS One. 2016 Jun 14;11(6):e0157383. doi: 10.1371/journal.pone.0157383 (PMC4907434; doi:10.1371/journal.pone.0157383)
Supplement: S2 Table — Two-tailed t-tests were used to compare the H-indices obtained for each sample. In particular, pairwise comparisons were made between each pair of time points for a given variety (a) and between pairs of varieties at a given time point (b). Statistically significant differences are indicated with asterisks (*p < 0.1, **p < 0.05, and ***p < 0.01). (DOCX) [file pone.0157383.s002.docx]

**S2 Table**. **Pairwise comparisons between time points and varieties.**

**a)**

| **Wine Variety** | ***sAF*** | ***24hAF*** | ***sMLF*** | ***hMLF*** | ***eMLF*** |
| --- | --- | --- | --- | --- | --- |
| **Primitivo** |  |  |  |  |  |
| ***sAF*** |  | ***** | *** | **** | ***** |
| ***24hAF*** | ***** |  | ***** | **** | ***** |
| ***sMLF*** | **** | ***** |  | **** | ***** |
| ***hMLF*** | **** | **** | **** |  |  |
| ***eMLF*** | ***** | ***** | ***** |  |  |
| **Negramaro** |  |  |  |  |  |
| ***sAF*** |  | ***** | ***** |  | ***** |
| ***24hAF*** | ***** |  | ***** | **** | ***** |
| ***sMLF*** | ***** | ***** |  |  | ***** |
| ***hMLF*** |  | **** |  |  | **** |
| ***eMLF*** | ***** | ***** | ***** | **** |  |
| **Cabernet** |  |  |  |  |  |
| ***sAF*** |  | **** |  | **** | ***** |
| ***24hAF*** | **** |  | **** | **** | ***** |
| ***sMLF*** |  | **** |  | **** | ***** |
| ***hMLF*** | **** | **** | **** |  | *** |
| ***eMLF*** | ***** | ***** | ***** | *** |  |

**b)**

| **Time point** | **Cabernet** | **Negramaro** | **Primitivo** |
| --- | --- | --- | --- |
| ***sAF*** |  |  |  |
| **Cabernet** |  | ***** |  |
| **Negramaro** | ***** |  | ***** |
| **Primitivo** |  | ***** |  |
| ***24hAF*** |  |  |  |
| Cabernet |  | ***** | **** |
| Negramaro | ***** |  | ***** |
| Primitivo | **** | ***** |  |
| ***sMLF*** |  |  |  |
| Cabernet |  |  | *** |
| Negramaro |  |  |  |
| Primitivo | *** |  |  |
| ***hMLF*** |  |  |  |
| Cabernet |  |  |  |
| Negramaro |  |  |  |
| Primitivo |  |  |  |
| ***eMLF*** |  |  |  |
| Cabernet |  | *** |  |
| Negramaro | *** |  | **** |
