# Supplementary material for: Complexity and Dynamics of the Winemaking Bacterial Communities in Berries, Musts, and Wines from Apulian Grape Cultivars through Time and Space
Source: PLoS One. 2016 Jun 14;11(6):e0157383. doi: 10.1371/journal.pone.0157383 (PMC4907434; doi:10.1371/journal.pone.0157383)
Supplement: S3 Table — Data related to the obtained taxonomic classification. In particular, (i) Sample Name = label assigned to the analysed sample, (ii) Classified = number of classified paired-end (PE) reads, (iii) Kingdom: PE reads classified at the kingdom level, (iv) % Kingdom: percentage of PE reads assigned at the kingdom level, relative to the total number of assigned PE reads, (v) Class: PE reads classified at the class level, (vi) % Class: percentage of PE reads assigned at the class level, relative to the total number of assigned PE reads, (vii) Order: PE reads classified at the order level, (viii) % Order: percentage of PE reads assigned at the order level, relative to the total number of assigned PE reads, (ix) Family: PE reads classified at the family level, (x) % Family: percentage of PE reads assigned at the family level, relative to the total number of assigned PE reads, (xi) Genus: PE reads classified at the genus level, (xii) % Genus: percentage of PE reads assigned at the genus level, relative to the total number of assigned PE reads, (xiii) Species: PE reads classified at the species level, (xiv) % Species: percentage of PE reads assigned at the species level, relative to the total number of assigned PE reads. (DOCX) [file pone.0157383.s003.docx]

**S2 Table**. **Taxonomic assignment analysis performed by BioMaS**.

| *Sample Name* | *Classified* | *Kingdom* | *% Kingdom* | *Phylum* | *% Phylum* | *Order* | *% Order* | *Class* | *% Class* | *Family* | *% Family* | *Genus* | *%Genus* | *Species* | *% Species* |
| --- | --- | --- | --- | --- | --- | --- | --- | --- | --- | --- | --- | --- | --- | --- | --- |
| *C_sAF_A_1* | *80,712* | *80,712* | *100.00%* | *80,712* | *100.00%* | *72,671* | *90.04%* | *77,218* | *95.67%* | *70,677* | *87.57%* | *75,918* | *94.06%* | *40,600* | *50.30%* |
| *C_sAF_A_2* | *111,645* | *111,645* | *100.00%* | *111,645* | *100.00%* | *103,775* | *92.95%* | *108,098* | *96.82%* | *101,892* | *91.26%* | *106,897* | *95.75%* | *61,241* | *54.85%* |
| *C_24hAF_A_1* | *99,424* | *99,424* | *100.00%* | *99,424* | *100.00%* | *95,391* | *95.94%* | *98,240* | *98.81%* | *95,174* | *95.73%* | *97,591* | *98.16%* | *66,462* | *66.85%* |
| *C_24hAF_A_2* | *102,509* | *102,509* | *100.00%* | *102,509* | *100.00%* | *97,006* | *94.63%* | *101,084* | *98.61%* | *96,766* | *94.40%* | *100,164* | *97.71%* | *67,498* | *65.85%* |
| *C_sMLF_A_1* | *150,771* | *150,771* | *100.00%* | *150,771* | *100.00%* | *145,640* | *96.60%* | *149,830* | *99.38%* | *145,529* | *96.52%* | *149,088* | *98.88%* | *115,105* | *76.34%* |
| *C_sMLF_A_2* | *127,641* | *127,641* | *100.00%* | *127,641* | *100.00%* | *120,518* | *94.42%* | *126,024* | *98.73%* | *120,336* | *94.28%* | *124,960* | *97.90%* | *94,628* | *74.14%* |
| *C_hMLF_A_1* | *372,625* | *372,625* | *100.00%* | *372,625* | *100.00%* | *372,582* | *99.99%* | *372,602* | *99.99%* | *372,475* | *99.96%* | *372,474* | *99.96%* | *357,228* | *95.87%* |
| *C_hMLF_A_2* | *197,879* | *197,879* | *100.00%* | *197,879* | *100.00%* | *197,715* | *99.92%* | *197,760* | *99.94%* | *197,602* | *99.86%* | *197,665* | *99.89%* | *186,371* | *94.18%* |
| *C_eMLF_A_1* | *268,519* | *268,519* | *100.00%* | *268,519* | *100.00%* | *268,496* | *99.99%* | *268,507* | *100.00%* | *268,493* | *99.99%* | *268,501* | *99.99%* | *257,942* | *96.06%* |
| *C_eMLF_A_2* | *358,774* | *358,774* | *100.00%* | *358,774* | *100.00%* | *358,744* | *99.99%* | *358,762* | *100.00%* | *358,693* | *99.98%* | *358,708* | *99.98%* | *344,862* | *96.12%* |
| *N_sAF_A_1* | *47,855* | *47,855* | *100.00%* | *47,855* | *100.00%* | *43,393* | *90.68%* | *45,811* | *95.73%* | *42,214* | *88.21%* | *45,175* | *94.40%* | *25,349* | *52.97%* |
| *N_sAF_A_2* | *55,181* | *55,181* | *100.00%* | *55,181* | *100.00%* | *53,549* | *97.04%* | *53,983* | *97.83%* | *52,685* | *95.48%* | *53,732* | *97.37%* | *34,424* | *62.38%* |
| *N_24hAF_A_1* | *197,727* | *197,727* | *100.00%* | *197,727* | *100.00%* | *196,417* | *99.34%* | *196,756* | *99.51%* | *195,875* | *99.06%* | *196,343* | *99.30%* | *131,965* | *66.74%* |
| *N_24hAF_A_2* | *208,596* | *208,596* | *100.00%* | *208,596* | *100.00%* | *206,079* | *98.79%* | *207,187* | *99.32%* | *205,292* | *98.42%* | *206,520* | *99.00%* | *155,837* | *74.71%* |
| *N_sMLF_A_1* | *100,138* | *100,138* | *100.00%* | *100,138* | *100.00%* | *96,335* | *96.20%* | *99,431* | *99.29%* | *96,205* | *96.07%* | *98,867* | *98.73%* | *77,970* | *77.86%* |
| *N_sMLF_A_2* | *145,597* | *145,597* | *100.00%* | *145,597* | *100.00%* | *142,759* | *98.05%* | *144,923* | *99.54%* | *142,567* | *97.92%* | *144,468* | *99.22%* | *120,690* | *82.89%* |
| *N_hMLF_A_1* | *346,144* | *346,144* | *100.00%* | *346,144* | *100.00%* | *346,049* | *99.97%* | *346,108* | *99.99%* | *345,946* | *99.94%* | *346,017* | *99.96%* | *330,975* | *95.62%* |
| *N_hMLF_A_2* | *332,736* | *332,736* | *100.00%* | *332,736* | *100.00%* | *332,647* | *99.97%* | *332,632* | *99.97%* | *331,734* | *99.70%* | *331,997* | *99.78%* | *311,106* | *93.50%* |
| *N_eMLF_A_1* | *347,704* | *347,704* | *100.00%* | *347,704* | *100.00%* | *347,675* | *99.99%* | *347,694* | *100.00%* | *347,662* | *99.99%* | *347,682* | *99.99%* | *333,296* | *95.86%* |
| *N_eMLF_A_2* | *316,365* | *316,365* | *100.00%* | *316,365* | *100.00%* | *316,296* | *99.98%* | *316,351* | *100.00%* | *316,269* | *99.97%* | *316,314* | *99.98%* | *304,972* | *96.40%* |
| *P_sAF_A_1* | *133,246* | *133,246* | *100.00%* | *133,246* | *100.00%* | *128,413* | *96.37%* | *131,197* | *98.46%* | *126,797* | *95.16%* | *129,769* | *97.39%* | *57,457* | *43.12%* |
| *P_sAF_A_2* | *68,141* | *68,141* | *100.00%* | *68,141* | *100.00%* | *63,210* | *92.76%* | *65,534* | *96.17%* | *61,567* | *90.35%* | *64,934* | *95.29%* | *36,962* | *54.24%* |
| *P_24sAF_A_1* | *187,035* | *187,035* | *100.00%* | *187,035* | *100.00%* | *184,535* | *98.66%* | *186,425* | *99.67%* | *184,328* | *98.55%* | *185,641* | *99.25%* | *105,766* | *56.55%* |
| *P_24hAF_A_2* | *259,012* | *259,012* | *100.00%* | *259,012* | *100.00%* | *255,768* | *98.75%* | *258,362* | *99.75%* | *255,499* | *98.64%* | *257,699* | *99.49%* | *185,994* | *71.81%* |
| *P_SMLF_A_1* | *199,913* | *199,913* | *100.00%* | *199,913* | *100.00%* | *197,381* | *98.73%* | *199,428* | *99.76%* | *197,352* | *98.72%* | *199,006* | *99.55%* | *136,046* | *68.05%* |
| *P_sMLF_A_2* | *166,951* | *166,951* | *100.00%* | *166,951* | *100.00%* | *163,067* | *97.67%* | *166,103* | *99.49%* | *162,809* | *97.52%* | *165,402* | *99.07%* | *138,463* | *82.94%* |
| *P_hMLF_A_1* | *412,777* | *412,777* | *100.00%* | *412,777* | *100.00%* | *412,668* | *99.97%* | *412,740* | *99.99%* | *412,647* | *99.97%* | *412,697* | *99.98%* | *392,549* | *95.10%* |
| *P_hMLF_A_2* | *306,588* | *306,588* | *100.00%* | *306,588* | *100.00%* | *306,495* | *99.97%* | *306,553* | *99.99%* | *306,328* | *99.92%* | *306,360* | *99.93%* | *290,981* | *94.91%* |
| *P_eMLF_A_1* | *291,729* | *291,729* | *100.00%* | *291,729* | *100.00%* | *291,662* | *99.98%* | *291,715* | *100.00%* | *291,646* | *99.97%* | *291,688* | *99.99%* | *276,061* | *94.63%* |
| *P_eMLF_A_2* | *453,582* | *453,582* | *100.00%* | *453,582* | *100.00%* | *453,533* | *99.99%* | *453,566* | *100.00%* | *453,515* | *99.99%* | *453,536* | *99.99%* | *434,268* | *95.74%* |
| *C_sAF_B_1* | *76,668* | *76,668* | *100.00%* | *76,668* | *100.00%* | *66,503* | *86.74%* | *73,217* | *95.50%* | *64,721* | *84.42%* | *71,404* | *93.13%* | *35,947* | *46.89%* |
| *C_sAF_B_2* | *100,810* | *100,810* | *100.00%* | *100,810* | *100.00%* | *91,529* | *90.79%* | *97,452* | *96.67%* | *89,870* | *89.15%* | *95,917* | *95.15%* | *53,229* | *52.80%* |
| *C_24hAF_B_1* | *90,079* | *90,079* | *100.00%* | *90,079* | *100.00%* | *85,299* | *94.69%* | *88,800* | *98.58%* | *85,043* | *94.41%* | *87,632* | *97.28%* | *58,887* | *65.37%* |
| *C_24hAF_B_2* | *110,387* | *110,387* | *100.00%* | *110,387* | *100.00%* | *103,064* | *93.37%* | *108,653* | *98.43%* | *102,748* | *93.08%* | *107,270* | *97.18%* | *70,790* | *64.13%* |
| *C_sMLF_B_1* | *140,464* | *140,464* | *100.00%* | *140,464* | *100.00%* | *133,938* | *95.35%* | *139,387* | *99.23%* | *133,773* | *95.24%* | *138,244* | *98.42%* | *104,983* | *74.74%* |
| *C_sMLF_B_2* | *116,132* | *116,132* | *100.00%* | *116,132* | *100.00%* | *107,579* | *92.64%* | *114,375* | *98.49%* | *107,302* | *92.40%* | *112,842* | *97.17%* | *82,646* | *71.17%* |
| *C_hMLF_B_1* | *343,114* | *343,114* | *100.00%* | *343,114* | *100.00%* | *343,073* | *99.99%* | *343,071* | *99.99%* | *342,945* | *99.95%* | *342,957* | *99.95%* | *322,398* | *93.96%* |
| *C_hMLF_B_2* | *318,806* | *318,806* | *100.00%* | *318,806* | *100.00%* | *318,553* | *99.92%* | *318,647* | *99.95%* | *318,424* | *99.88%* | *318,512* | *99.91%* | *296,089* | *92.87%* |
| *C_eMLF_B_1* | *229,131* | *229,131* | *100.00%* | *229,131* | *100.00%* | *229,089* | *99.98%* | *229,121* | *100.00%* | *229,078* | *99.98%* | *229,101* | *99.99%* | *215,821* | *94.19%* |
| *C_eMLF_B_2* | *325,529* | *325,529* | *100.00%* | *325,529* | *100.00%* | *325,479* | *99.98%* | *325,511* | *99.99%* | *325,437* | *99.97%* | *325,465* | *99.98%* | *306,866* | *94.27%* |
| *N_sAF_B_1* | *42,651* | *42,651* | *100.00%* | *42,651* | *100.00%* | *37,249* | *87.33%* | *40,734* | *95.51%* | *36,141* | *84.74%* | *39,677* | *93.03%* | *21,400* | *50.17%* |
| *N_sAF_B_2* | *53,584* | *53,584* | *100.00%* | *53,584* | *100.00%* | *51,200* | *95.55%* | *52,382* | *97.76%* | *50,357* | *93.98%* | *51,684* | *96.45%* | *31,879* | *59.49%* |
| *N_24hAF_B_1* | *177,651* | *177,651* | *100.00%* | *177,651* | *100.00%* | *176,164* | *99.16%* | *176,773* | *99.51%* | *175,671* | *98.89%* | *176,339* | *99.26%* | *115,777* | *65.17%* |
| *N_24hAF_B_2* | *198,193* | *198,193* | *100.00%* | *198,193* | *100.00%* | *195,206* | *98.49%* | *196,890* | *99.34%* | *194,421* | *98.10%* | *196,097* | *98.94%* | *145,165* | *73.24%* |
| *N_sMLF_B_1* | *85,189* | *85,189* | *100.00%* | *85,189* | *100.00%* | *80,625* | *94.64%* | *84,455* | *99.14%* | *80,474* | *94.47%* | *83,709* | *98.26%* | *64,466* | *75.67%* |
| *N_sMLF_B_2* | *143,858* | *143,858* | *100.00%* | *143,858* | *100.00%* | *139,570* | *97.02%* | *143,074* | *99.46%* | *139,348* | *96.86%* | *142,247* | *98.88%* | *112,876* | *78.46%* |
| *N_hMLF_B_1* | *312,915* | *312,915* | *100.00%* | *312,915* | *100.00%* | *312,773* | *99.95%* | *312,860* | *99.98%* | *312,679* | *99.92%* | *312,768* | *99.95%* | *293,117* | *93.67%* |
| *N_hMLF_B_2* | *292,030* | *292,030* | *100.00%* | *292,030* | *100.00%* | *291,924* | *99.96%* | *291,935* | *99.97%* | *291,144* | *99.70%* | *291,333* | *99.76%* | *267,372* | *91.56%* |
| *N_eMLF_B_1* | *296,095* | *296,095* | *100.00%* | *296,095* | *100.00%* | *296,061* | *99.99%* | *296,076* | *99.99%* | *296,055* | *99.99%* | *296,071* | *99.99%* | *278,147* | *93.94%* |
| *N_eMLF_B_2* | *289,306* | *289,306* | *100.00%* | *289,306* | *100.00%* | *289,182* | *99.96%* | *289,269* | *99.99%* | *289,140* | *99.94%* | *289,209* | *99.97%* | *273,038* | *94.38%* |
| *P_sAF_B_1* | *112,045* | *112,045* | *100.00%* | *112,045* | *100.00%* | *106,538* | *95.09%* | *110,231* | *98.38%* | *105,246* | *93.93%* | *108,841* | *97.14%* | *46,518* | *41.52%* |
| *P_sAF_B_2* | *63,994* | *63,994* | *100.00%* | *63,994* | *100.00%* | *57,895* | *90.47%* | *61,405* | *95.95%* | *56,185* | *87.80%* | *60,536* | *94.60%* | *32,947* | *51.48%* |
| *P_24sAF_B_1* | *162,654* | *162,654* | *100.00%* | *162,654* | *100.00%* | *159,754* | *98.22%* | *161,928* | *99.55%* | *159,503* | *98.06%* | *160,952* | *98.95%* | *88,945* | *54.68%* |
| *P_24hAF_B_2* | *241,985* | *241,985* | *100.00%* | *241,985* | *100.00%* | *237,053* | *97.96%* | *241,035* | *99.61%* | *236,713* | *97.82%* | *239,935* | *99.15%* | *167,654* | *69.28%* |
| *P_SMLF_B_1* | *179,018* | *179,018* | *100.00%* | *179,018* | *100.00%* | *176,073* | *98.35%* | *178,533* | *99.73%* | *176,013* | *98.32%* | *178,028* | *99.45%* | *119,251* | *66.61%* |
| *P_sMLF_B_2* | *150,672* | *150,672* | *100.00%* | *150,672* | *100.00%* | *145,383* | *96.49%* | *149,838* | *99.45%* | *145,176* | *96.35%* | *148,853* | *98.79%* | *122,369* | *81.22%* |
| *P_hMLF_B_1* | *377,161* | *377,161* | *100.00%* | *377,161* | *100.00%* | *377,054* | *99.97%* | *377,119* | *99.99%* | *377,016* | *99.96%* | *377,065* | *99.97%* | *352,910* | *93.57%* |
| *P_hMLF_B_2* | *273,603* | *273,603* | *100.00%* | *273,603* | *100.00%* | *273,525* | *99.97%* | *273,571* | *99.99%* | *273,384* | *99.92%* | *273,413* | *99.93%* | *254,990* | *93.20%* |
| *P_eMLF_B_1* | *270,560* | *270,560* | *100.00%* | *270,560* | *100.00%* | *270,476* | *99.97%* | *270,543* | *99.99%* | *270,462* | *99.96%* | *270,509* | *99.98%* | *252,148* | *93.19%* |
| *P_eMLF_B_2* | *436,279* | *436,279* | *100.00%* | *436,279* | *100.00%* | *436,248* | *99.99%* | *436,270* | *100.00%* | *436,232* | *99.99%* | *436,246* | *99.99%* | *412,143* | *94.47%* |
