# Supplementary material for: Complexity and Dynamics of the Winemaking Bacterial Communities in Berries, Musts, and Wines from Apulian Grape Cultivars through Time and Space
Source: PLoS One. 2016 Jun 14;11(6):e0157383. doi: 10.1371/journal.pone.0157383 (PMC4907434; doi:10.1371/journal.pone.0157383)
Supplement: S4 Table — List of taxa at the genus and species levels identified in the Cabernet, Negramaro, and Primitivo samples that were not detected in the field (the bww samples) but were present during fermentation from start (sAF) to finish (eMLF). (DOCX) [file pone.0157383.s004.docx]

**S3 Table**. **Winery microflora associated with the tested wine varieties.**

| ***Variety*** | ***Genus*** | ***Species*** |
| --- | --- | --- |
|  | *Micrococcus* | *Acetobacter_sp._150* |
| Cabernet |  | *Brevundimonas_bullata* |
|  |  | *Sphingomonas_sp._CS81* |
|  | *Haemophilus* | *Acetobacter_sp._3-C-3* |
|  | *Luteimonas* | *Acidovorax_sp._BJC8* |
|  | *Wautersiella* | *Acinetobacter_sp._SOD-103* |
|  |  | *Bacillus_sp._YDWLR1* |
| Negramaro |  | *Clostridiales_Family_XI._Incertae_Sedis* |
|  |  | *Comamonas_aquatica* |
|  |  | *Haemophilus_parainfluenzae* |
|  |  | *Pseudomonas_plecoglossicida* |
|  |  | *Sphingomonas_sp._MG-2011-115-FX* |
|  |  | *Streptococcus_sanguinis* |
|  | *Actinomyces* | *Acetobacter_cerevisiae* |
|  | *Alistipes* | *Aeromonas_sp._symbiont_of_Sitobion_miscanthi* |
|  | *Ameyamaea* | *Arthrobacter_sp._MDB1-56* |
|  | *Aurantimonas* | *Bacillus_pichinotyi* |
|  | *Caenimonas* | *Empedobacter_sp._F-Fue-04IIIbab* |
|  | *Cellvibrio* | *Empedobacter_sp._F-Fue-04TIIa* |
|  | *Dietzia* | *Enterococcus_faecalis* |
|  | *Empedobacter* | *Geodermatophilus_tzadiensis* |
|  | *Leifsonia* | *Gluconobacter_sp._aP112* |
|  | *Micrococcus* | *Hymenobacter_ginsengisoli* |
|  | *Nevskia* | *Novosphingobium_sp._SaCR2* |
| Primitivo | *Pediococcus* | *Paenibacillus_sp._JN20* |
|  | *Ruminococcus* | *Paenibacillus_taichungensis* |
|  | *Tolumonas* | *Pantoea_sp._P10QLC* |
|  | *Tsukamurella* | *Pseudomonas_palleroniana* |
|  | *Wautersiella* | *Pseudomonas_sabulinigri* |
|  |  | *Pseudomonas_sp._Cantas10* |
|  |  | *Pseudomonas_sp._MSSRFD87* |
|  |  | *Pseudomonas_tolaasii* |
|  |  | *Rahnella_sp._I_Gauze_A_4_5* |
|  |  | *Serratia_proteamaculans* |
|  |  | *Sphingomonas_sp._ce29* |
|  |  | *Sphingomonas_sp._IMER-A2-12* |
|  |  | *Staphylococcus_saprophyticus* |
|  |  | *Staphylococcus_sp._SDT18* |
|  |  | *Tolumonas_auensis* |
|  |  | *Variovorax_paradoxus* |
|  |  | *Wautersiella_sp._M1T8B12* |
