# Supplementary material for: Complexity and Dynamics of the Winemaking Bacterial Communities in Berries, Musts, and Wines from Apulian Grape Cultivars through Time and Space
Source: PLoS One. 2016 Jun 14;11(6):e0157383. doi: 10.1371/journal.pone.0157383 (PMC4907434; doi:10.1371/journal.pone.0157383)
Supplement: S5 Table — List of the species that originated in the field and persisted throughout the fermentation process, from start (sAF) to finish (eMLF). (DOCX) [file pone.0157383.s005.docx]

**S4 Table. List of the species that originated in the field and endured along the entire fermentation process**

| Variety | Species |
| --- | --- |
| Cabernet | *Acetobacter_aceti, Acetobacter_cerevisiae, Acetobacter_cibinongensis, Acetobacter_estunensis, Acetobacter_indonesiensis, Acetobacter_lovaniensis, Acetobacter_malorum, Acetobacter_nitrogenifigens, Acetobacter_oeni, Acetobacter_orleanensis, Acetobacter_pasteurianus, Acetobacter_peroxydans, Acetobacter_sp._3-C3, Acetobacter_tropicalis, Acidovorax_valerianellae, Acinetobacter_baumannii, Acinetobacter_calcoaceticus, Acinetobacter_johnsonii, Acinetobacter_junii, Acinetobacter_lwoffii, Acinetobacter_sp._1g, Acinetobacter_sp._31, Acinetobacter_sp._6_2012_, Acinetobacter_sp._EKI16, Acinetobacter_sp._JH25010, Acinetobacter_sp._Ld2, Acinetobacter_sp._PMP1, Acinetobacter_sp._SOD103, Acinetobacter_sp._TN302, Acinetobacter_sp._TPN102, Acinetobacter_sp._XA05, Ameyamaea_chiangmaiensis, Arctic_sea_ice_bacterium_ARK10033, Arthrobacter_globiformis, Bacillus_oshimensis, Bacillus_sp._NR7_UPM, Belnapia_moabensis, Bryocella_elongata, Chelativorans_sp._B0.0998, Citrobacter_freundii, Comamonas_aquatica, Comamonas_sp._WT_OTU1, Commensalibacter_intestini, Curtobacterium_flaccumfaciens, Curtobacterium_sp._D34, Curvibacter_delicatus, Delftia_tsuruhatensis, Enterobacter_aerogenes, Enterobacter_asburiae, Enterobacter_soli, Enterobacter_sp._23R_G03, Enterobacter_sp._BIHB_1401, Enterobacter_sp._Hg402, Enterobacter_sp._M4, Enterobacter_sp._MK33, Enterobacter_sp._TN468, Erwinia_billingiae, Flexibacter_sp._MG5, Gilliamella_apicola, Gluconacetobacter_entanii, Gluconacetobacter_persimmonis, Gluconacetobacter_sp._Gs4, Gluconacetobacter_sp._RKY5, Gluconobacter_albidus, Gluconobacter_cerinus, Gluconobacter_frateurii, Gluconobacter_kanchanaburiensis, Gluconobacter_oxydans, Gluconobacter_sp._NBRC_3243, Gluconobacter_sp._TMW_2.767, Gluconobacter_wancherniae, Halomonas_phoceae, Halomonas_rifensis, Halomonas_sp._A-3, Halomonas_sp._A7, Hymenobacter_aerophilus, Hymenobacter_ginsengisoli, Hymenobacter_sp._R36499, Klebsiella_oxytoca, Klebsiella_sp._E8.2, Klebsiella_sp._GG50E, Klebsiella_sp._MX, Klebsiella_sp._RV_F03_18c, Komagataeibacter_europaeus, Komagataeibacter_hansenii, Komagataeibacter_intermedius, Komagataeibacter_kakiaceti, Komagataeibacter_maltaceti, Komagataeibacter_medellinensis, Komagataeibacter_oboediens, Komagataeibacter_rhaeticus, Komagataeibacter_saccharivorans, Komagataeibacter_sucrofermentans, Komagataeibacter_xylinus, Kozakia_baliensis, Lactobacillus_brevis, Lactobacillus_collinoides, Lactobacillus_mali, Lactobacillus_paracollinoides, Lactobacillus_plantarum, Lactobacillus_sp._RMS31, Lactobacillus_sp._T3R2C12, Lactococcus_lactis, Lactococcus_sp._MH52, Lelliottia_amnigena, Leuconostoc_gasicomitatum, Leuconostoc_lactis, Leuconostoc_mesenteroides, Lysinibacillus_fusiformis, Marmoricola_aurantiacus, Massilia_sp._4D3c, Methylobacterium_adhaesivum, Methylobacterium_dankookense, Methylobacterium_extorquens, Methylobacterium_fujisawaense, Methylobacterium_longum, Methylobacterium_mesophilicum, Methylobacterium_populi, Methylobacterium_radiotolerans, Methylobacterium_rhodesianum, Methylobacterium_sp._104, Methylobacterium_sp._3R6, Methylobacterium_sp._AR1.6/1, Methylobacterium_sp._ccc_42, Methylobacterium_sp._II_GA_A_1_8, Methylobacterium_sp._nac_6, Methylobacterium_sp._nac_9, Methylobacterium_sp._nah_6, Methylobacterium_sp._NBCS22, Methylobacterium_sp._PB23, Methylobacterium_sp._sc_3, Methylobacterium_sp._XJLW, Microbacterium_sp._CNJ797_PL04, Microbacterium_sp._enrichment_culture_clone_GR9, Microbacterium_sp._MSB14, Microbacterium_sp._S18, Naxibacter_sp._AF_NAK1-3, Nocardioides_sp._BA32_2011_, Oenococcus_oeni, Oxalobacter_sp._P138, Paenibacillus_pabuli, Pantoea_agglomerans, Pantoea_ananatis, Pantoea_rwandensis, Pantoea_septica, Pantoea_sp._3038, Pantoea_sp._B3071, Pantoea_sp._EM486, Pantoea_sp._S97, Pantoea_sp._symbiont_of_Sitobion_miscanthi, Pantoea_stewartii, Pantoea_vagans, Pectobacterium_carotovorum, Pediococcus_pentosaceus, Pedobacter_sp._JC2492, Propionibacterium_acnes, Propionibacterium_sp._MSeoKT2, Propionibacterium_sp._Ob_aa_02, Pseudomonas_chlororaphis, Pseudomonas_putida, Pseudomonas_sabulinigri, Pseudomonas_sp._EMB_1, Pseudomonas_sp._PCZ05H, Pseudomonas_sp._ps104, Rahnella_aquatilis, Ralstonia_solanacearum, Ralstonia_sp._PHS1, Ralstonia_syzygii, Raoultella_ornithinolytica, Raoultella_planticola, Roseomonas_aerilata, Roseomonas_sp._UAPS01001, Serratia_proteamaculans, Shewanella_algae, Shewanella_aquimarina, Shewanella_baltica, Shewanella_halifaxensis, Shewanella_haliotis, Shewanella_pealeana, Shewanella_putrefaciens, Shewanella_sp._17.3_KSS, Shewanella_sp._a111-132, Shewanella_sp._A317, Shewanella_sp._ANA3, Shewanella_sp._enrichment_culture_clone_KWE3029, Shewanella_sp._KTSW-7, Shewanella_sp._MA342, Shewanella_sp._MA357, Shewanella_sp._MCCB_144, Shewanella_sp._MR4, Shewanella_sp._SG133, Shewanella_sp._VA_C1-5, Shewanella_sp._Zh23, Sphingomonas_aerolata, Sphingomonas_aquatilis, Sphingomonas_endophytica, Sphingomonas_insulae, Sphingomonas_melonis, Sphingomonas_mucosissima, Sphingomonas_phyllosphaerae, Sphingomonas_sp._17, Sphingomonas_sp._75, Sphingomonas_sp._DS18, Sphingomonas_sp._Es14, Sphingomonas_sp._IK2_77, Sphingomonas_sp._IW3, Sphingomonas_sp._K6D38P, Sphingomonas_sp._M323, Sphingomonas_sp._nac_25, Sphingomonas_sp._nah_25, Sphingomonas_sp._PFI, Sphingomonas_sp._SOZ1-3091, Sphingomonas_sp._TNR-2, Sphingomonas_sp._W2.1027, Sphingomonas_yunnanensis, Staphylococcus_epidermidis, Staphylococcus_sp._C16-Siri111, Staphylococcus_sp._DV9-14, Stenotrophomonas_maltophilia, Stenotrophomonas_rhizophila, Tanticharoenia_sakaeratensis, Terriglobus_sp._enrichment_culture_clone_03SUJ4, Tolumonas_auensis, uncultured_Acidobacteriales_bacterium, uncultured_actinobacterium, uncultured_proteobacterium, Variovorax_paradoxus, Wolbachia_endosymbiont_of_Bemisia_tabaci, Xanthomonas_sp._PHCDB5* |
| Negramaro | *Acetobacter_aceti, Acetobacter_indonesiensis, Acetobacter_malorum, Acetobacter_oeni, Acetobacter_pasteurianus, Acetobacter_tropicalis, Acidovorax_valerianellae, Acinetobacter_baumannii, Acinetobacter_johnsonii, Acinetobacter_lwoffii, Acinetobacter_sp._1g, Acinetobacter_sp._6_2012_, Acinetobacter_sp._EK-I16, Acinetobacter_sp._JH25010, Acinetobacter_sp._Ld2, Acinetobacter_sp._TPN102, Acinetobacter_sp._XA05, Afipia_genosp._1, Arctic_sea_ice_bacterium_ARK10033, Belnapia_moabensis, Bradyrhizobium_elkanii, Burkholderia_fungorum, Citrobacter_freundii, Cloacibacterium_normanense, Comamonas_sp._WT_OTU1, Commensalibacter_intestini, Curtobacterium_sp._D34, Delftia_tsuruhatensis, Enterobacter_asburiae, Enterobacter_sp._23R_G03, Enterobacter_sp._638, Enterobacter_sp._Hg402, Enterobacter_sp._MK33, Enterobacter_sp._RV_G03_19c, Enterobacter_sp._YUST-DW21, Erwinia_billingiae, Erwinia_sp._enrichment_culture_clone_MRHull-S11H, Escherichia_coli, Escherichia_hermannii, Gibbsiella_dentisursi, Gilliamella_apicola, Gluconobacter_albidus, Gluconobacter_cerinus, Gluconobacter_frateurii, Gluconobacter_oxydans, Gluconobacter_sp._NBRC_3243, Gluconobacter_wancherniae, Halomonas_phoceae, Halomonas_rifensis, Halomonas_sp._A-3, Halomonas_sp._A-7, Halomonas_sp._A8, Halomonas_sp._ljh-38, Klebsiella_oxytoca, Klebsiella_sp._E8.2, Klebsiella_sp._GG50E, Klebsiella_sp._MX, Klebsiella_sp._RV_F03_18c, Komagataeibacter_europaeus, Komagataeibacter_hansenii, Komagataeibacter_kakiaceti, Komagataeibacter_medellinensis, Komagataeibacter_xylinus, Lactobacillus_murinus, Lelliottia_amnigena, Leuconostoc_mesenteroides, Leuconostoc_pseudomesenteroides, Methylobacterium_dankookense, Methylobacterium_extorquens, Methylobacterium_fujisawaense, Methylobacterium_longum, Methylobacterium_mesophilicum, Methylobacterium_populi, Methylobacterium_rhodesianum, Methylobacterium_sp._104, Methylobacterium_sp._nac_6, Methylobacterium_sp._nac_9, Methylobacterium_sp._NBCS22, Methylobacterium_sp._PB23, Methylobacterium_sp._XJLW, Oenococcus_oeni, Pantoea_agglomerans, Pantoea_ananatis, Pantoea_dispersa, Pantoea_rwandensis, Pantoea_septica, Pantoea_sp._3038, Pantoea_sp._B3071, Pantoea_sp._E112, Pantoea_sp._P10QLC, Pantoea_sp._S97, Pantoea_sp._symbiont_of_Sitobion_miscanthi, Pantoea_sp._Ward, Pantoea_stewartii, Pectobacterium_carotovorum, Pluralibacter_pyrinus, Propionibacterium_acnes, Propionibacterium_sp._MSeoKT2, Propionibacterium_sp._Ob_aa_02, Pseudomonas_sp._PCZ05H, Pseudomonas_sp._THt122, Pseudoxanthomonas_suwonensis, Rahnella_sp._I_Gauze_A_4_5, Ralstonia_solanacearum, Ralstonia_sp._PHS1, Ralstonia_syzygii, Raoultella_ornithinolytica, Raoultella_planticola, Salmonella_enterica, Serratia_marcescens, Shewanella_algae, Shewanella_aquimarina, Shewanella_baltica, Shewanella_halifaxensis, Shewanella_haliotis, Shewanella_pealeana, Shewanella_putrefaciens, Shewanella_sp._17.3_KSS, Shewanella_sp._a111132, Shewanella_sp._A317, Shewanella_sp._AM10, Shewanella_sp._ANA3, Shewanella_sp._ANG.309, Shewanella_sp._enrichment_culture_clone_KWE30-29, Shewanella_sp._KTSW7, Shewanella_sp._MA342, Shewanella_sp._MA357, Shewanella_sp._MCCB_144, Shewanella_sp._MR4, Shewanella_sp._S03, Shewanella_sp._SG133, Shewanella_sp._VA_C1-5, Shewanella_sp._YASM-24, Shewanella_sp._Zh23, Sphingomonas_astaxanthinifaciens, Sphingomonas_faeni, Sphingomonas_melonis, Sphingomonas_phyllosphaerae, Sphingomonas_sp._DS18, Sphingomonas_sp._L9462, Sphingomonas_sp._nac_25, Staphylococcus_epidermidis, Staphylococcus_sp._C16-Siri111, Staphylococcus_sp._CHNDP23, Staphylococcus_sp._DV914, Staphylococcus_sp._MSB11, Staphylococcus_warneri, Streptococcus_sp._oral_clone_CH016, Tanticharoenia_sakaeratensis, uncultured_Acidobacteriales_bacterium, uncultured_actinobacterium, uncultured_Bacteroidales_ba* |
| Primitivo | *Acetobacter_cibinongensis, Acetobacter_farinalis, Acetobacter_indonesiensis, Acetobacter_lovaniensis, Acetobacter_malorum, Acetobacter_oeni, Acetobacter_orleanensis, Acetobacter_pasteurianus, Acetobacter_peroxydans, Acetobacter_persici, Acetobacter_sp., Acetobacter_sp._3-C3, Acetobacter_syzygii, Acetobacter_tropicalis, Acidovorax_sp._BJC8, Acinetobacter_baumannii, Acinetobacter_calcoaceticus, Acinetobacter_johnsonii, Acinetobacter_lwoffii, Acinetobacter_sp._1g, Acinetobacter_sp._31, Acinetobacter_sp._51, Acinetobacter_sp._6_2012_, Acinetobacter_sp._EKI16, Acinetobacter_sp._JH25010, Acinetobacter_sp._Ld2, Acinetobacter_sp._PMP1, Acinetobacter_sp._SOD103, Acinetobacter_sp._TN302, Acinetobacter_sp._TPN102, Acinetobacter_sp._XA05, Aeromonas_hydrophila, Aeromonas_sp._Z2_T_TSA_34, Agrobacterium_tumefaciens, Arctic_sea_ice_bacterium_ARK10033, Arthrobacter_oxydans, Asaia_siamensis, Bacillus_gibsonii, Bacillus_sp._DB135_2010_, Bacillus_sp._YDWLR1, Bradyrhizobium_elkanii, Bradyrhizobium_sp._LmjDb2, Citrobacter_freundii, Citrobacter_murliniae, Cloacibacterium_normanense, Comamonas_aquatica, Comamonas_sp._WT_OTU1, Commensalibacter_intestini, Curtobacterium_flaccumfaciens, Curtobacterium_sp._D34, Delftia_acidovorans, Delftia_tsuruhatensis, Dyella_sp._CHNCT13, Dyella_sp._WH32, Enterobacter_aerogenes, Enterobacter_asburiae, Enterobacter_cancerogenus, Enterobacter_ludwigii, Enterobacter_soli, Enterobacter_sp._23R_G03, Enterobacter_sp._2N01, Enterobacter_sp._860, Enterobacter_sp._BIHB_1401, Enterobacter_sp._d416, Enterobacter_sp._Hg402, Enterobacter_sp._M4, Enterobacter_sp._MK33, Enterobacter_sp._RV_G03_19c, Enterobacter_sp._TN468, Enterobacter_sp._WAB1915, Enterobacter_sp._YUSTDW21, Enterococcus_gallinarum, Enterococcus_sp._RU07, Erwinia_persicina, Erwinia_sp._A36, Frateuria_aurantia, Frateuria_sp._ECK130, Frischella_perrara, Gilliamella_apicola, Gluconacetobacter_entanii, Gluconacetobacter_persimmonis, Gluconacetobacter_sp._'munehiro', Gluconacetobacter_sp._Gs4, Gluconacetobacter_sp._RKY5, Gluconobacter_albidus, Gluconobacter_cerinus, Gluconobacter_frateurii, Gluconobacter_japonicus, Gluconobacter_kanchanaburiensis, Gluconobacter_nephelii, Gluconobacter_oxydans, Gluconobacter_sp._aP78, Gluconobacter_sp._NBRC_3243, Gluconobacter_sp._TMW_2.767, Gluconobacter_sp._WK1G3, Gluconobacter_sphaericus, Gluconobacter_thailandicus, Gluconobacter_wancherniae, Halomonas_desiderata, Halomonas_phoceae, Halomonas_rifensis, Halomonas_sp._A-3, Halomonas_sp._A-7, Halomonas_sp._A-8, Halomonas_sp._ljh38, Hymenobacter_sp._1018, Klebsiella_oxytoca, Klebsiella_pneumoniae, Klebsiella_sp._E8.2, Klebsiella_sp._GG50E, Klebsiella_sp._HX149S, Klebsiella_sp._MX, Klebsiella_sp._PG122E, Klebsiella_sp._SOZ17042, Klebsiella_variicola, Komagataeibacter_europaeus, Komagataeibacter_hansenii, Komagataeibacter_intermedius, Komagataeibacter_kakiaceti, Komagataeibacter_maltaceti, Komagataeibacter_medellinensis, Komagataeibacter_nataicola, Komagataeibacter_oboediens, Komagataeibacter_rhaeticus, Komagataeibacter_saccharivorans, Komagataeibacter_sucrofermentans, Komagataeibacter_swingsii, Komagataeibacter_xylinus, Kosakonia_oryzae, Kozakia_baliensis, Lactobacillus_brevis, Lactobacillus_mali, Lactococcus_lactis, Lactococcus_sp._MH52, Leclercia_adecarboxylata, Lelliottia_amnigena, Leuconostoc_mesenteroides, Leuconostoc_pseudomesenteroides, Massilia_sp._TS1112T, Methylobacterium_adhaesivum, Methylobacterium_dankookense, Methylobacterium_extorquens, Methylobacterium_fujisawaense, Methylobacterium_mesophilicum, Methylobacterium_populi, Methylobacterium_rhodesianum, Methylobacterium_sp._104, Methylobacterium_sp._NBCS22, Methylobacterium_sp._sc_3, Methylobacterium_sp._XJLW, Morganella_morganii, Oenococcus_oeni, Paenibacillus_pabuli, Paenibacillus_tylopili, Pantoea_agglomerans, Pantoea_ananatis, Pantoea_eucalypti, Pantoea_rwandensis, Pantoea_sp._3038, Pantoea_sp._B3071, Pantoea_sp._E112, Pantoea_sp._S97, Pantoea_sp._symbiont_of_Sitobion_miscanthi, Pantoea_stewartii, Pantoea_vagans, Pectobacterium_carotovorum, Phytobacter_diazotrophicus, Propionibacterium_acnes, Propionibacterium_sp._MSeoKT2, Providencia_alcalifaciens, Pseudomonas_aeruginosa, Pseudomonas_plecoglossicida, Pseudomonas_pseudoalcaligenes, Pseudomonas_putida, Pseudomonas_rhizosphaerae, Pseudomonas_sp._CBMAI_468, Pseudomonas_sp._CTN2, Pseudomonas_sp._EMB_1, Pseudomonas_sp._enrichment_culture_clone_2, Pseudomonas_sp._IHB_B_3393, Pseudomonas_sp._LS1, Pseudomonas_sp._MY1402, Pseudomonas_sp._PCZ05H, Pseudomonas_sp._ps10-4, Pseudomonas_sp._ps6-35, Pseudomonas_sp._THt122, Pseudomonas_sp._X20, Pseudoxanthomonas_sp._Eza14, Pseudoxanthomonas_suwonensis, Rahnella_aquatilis, Ralstonia_solanacearum, Ralstonia_sp._PHS1, Ralstonia_syzygii, Raoultella_ornithinolytica, Raoultella_planticola, Shewanella_algae, Shewanella_amazonensis, Shewanella_aquimarina, Shewanella_baltica, Shewanella_halifaxensis, Shewanella_haliotis, Shewanella_japonica, Shewanella_pealeana, Shewanella_putrefaciens, Shewanella_sp._17.3_KSS, Shewanella_sp._62_2006b_, Shewanella_sp._a111-132, Shewanella_sp._A317, Shewanella_sp._ANA3, Shewanella_sp._ANG.309, Shewanella_sp._enrichment_culture_clone_KWE30-29, Shewanella_sp._KTSW7, Shewanella_sp._MA342, Shewanella_sp._MA357, Shewanella_sp._MCCB_144, Shewanella_sp._MR4, Shewanella_sp._S03, Shewanella_sp._SG133, Shewanella_sp._VA_C1-5, Shewanella_sp._YASM-24, Shewanella_sp._Zh23, Sphingomonas_aquatilis, Sphingomonas_endophytica, Sphingomonas_melonis, Sphingomonas_phyllosphaerae, Sphingomonas_sp._nac_25, Sphingomonas_sp._PDD-27b-1, Sphingomonas_sp._SOZ1-3091, Staphylococcus_aureus, Staphylococcus_epidermidis, Staphylococcus_sp._C16-Siri111, Staphylococcus_sp._CT21, Staphylococcus_sp._DV9-14, Stenotrophomonas_maltophilia, Tanticharoenia_sakaeratensis, uncultured_Bacteroidales_bacterium, unc* |
